# Entomopathogenic Fungi for Tick Control in Cattle Livestock From Mexico

**DOI:** 10.3389/ffunb.2021.657694

**Published:** 2021-04-30

**Authors:** Miguel Angel Alonso-Díaz, Agustín Fernández-Salas

**Affiliations:** Centro de Enseñanza, Investigación y Extension en Ganadería Tropical, Facultad de Medicina Veterinaria y Zootecnia, Universidad Nacional Autónoma de México, Martínez de la Torre, Mexico

**Keywords:** *Rhipicephalus microplus*, *Metarhizium*, *Beauveria*, bovines, biological control, ticks

## Abstract

Ticks are one of the main economic threats to the cattle industry worldwide affecting productivity, health and welfare. The need for alternative methods to control tick populations is prompted by the high prevalence of multiresistant tick strains to the main chemical acaricides and their ecological consequences. Biological control using entomopathogenic fungi (EPF) is one of the most promising alternative options. The objective of this paper is to review the use of EPF as an alternative control method against cattle ticks in Mexico. *Metarhizium anisopliae* sensu lato (s.l.) and *Beauveria bassiana* s.l. are the most studied EPF for the biological control of ticks in the laboratory and in the field, mainly against *Rhipicephalus microplus*; however, evaluations against other important cattle ticks such as *Amblyomma mixtum* and *R*. *annulatus*, are needed. A transdisciplinary approach is required to incorporate different types of tools, such as genomics, transcriptomics and proteomics in order to better understand the pathogenicity/virulence mechanism in EPF against ticks. Laboratory tests have demonstrated the EPF efficacy to control susceptible and resistant/multiresistant tick populations; whereas, field tests have shown satisfactory control efficiency of *M*. *anisopliae* s.l. against different stages of *R*. *microplus* when applied both on pasture and on cattle. Epidemiological aspects of ticks and environmental factors are considered as components that influence the acaricidal behavior of the EPF. Finally, considering all these aspects, some recommendations are proposed for the use of EPF in integrated control schemes for livestock ticks.

## Introduction

Food security is one of the main concerns worldwide, where cattle play a fundamental role in the supply of milk and meat (Falvey, 2015). Cattle production in Mexico is an activity of social and economic importance that is carried out throughout the national territory, occupying more than 110 million hectares, with 1.1 million registered livestock farms (SIAP (Servicio de Información Agroalimentaria y Pesquera), 2020). This large area of the national territory dedicated to livestock has an impact on the use of natural resources and can affect the quality and preservation of ecosystems (González-Padilla et al., 2019). Bovine livestock in the country has an inventory of 35.2 million cattle heads (SIAP (Servicio de Información Agroalimentaria y Pesquera), 2018), and is based mainly on direct grazing in extensive production systems (Castillo-Gallegos et al., 2005); where one of the main threats are ticks and the pathogens they transmit, affecting productivity, health and well-being. It has been estimated that more than 80% of cattle population worldwide is exposed to tick infestations (Snelson, 1975; Giles et al., 2014), where the cattle tick *Rhipicephalus* (*Boophilus*) *microplus* (Canestrini), *R*. (*B*.) *annulatus* (Say), and *Amblyomma mixtum* (Koch) are considered the most important livestock ticks in Mexico. Previously, tick control has been based on therapeutic interventions using chemical treatments (acaricides and endectocides). These methodologies have definitely contributed to improving productivity and welfare; however, the intensive and frequent use, and inappropriate use as well, of these chemicals has resulted in the development of acaricidal resistance in ticks (Fernández-Salas et al., 2012a,b,c; Alonso-Díaz et al., 2013a). Tick resistance has been reported for almost all the main chemical acaricides (Alonso-Díaz et al., 2013a; Rodríguez-Vivas et al., 2014a) and this phenomenon, added to an exacerbated chemical control problem, has had other consequences such as environmental and food contamination by secondary chemical metabolites, spread of ticks into free zones, restrictions on cattle export and increase in diseases transmitted by these parasites (De Castro, 1997; Domínguez-García et al., 2016; Rodríguez-Vivas et al., 2017). Results have shown that dependence on these chemical products, as the only form of control, is neither economically nor ecologically sustainable. Sustainable cattle production needs strong changes, such as considering both agroecology-oriented and novel tick-control approaches (Alonso-Díaz et al., 2014). This latter has motivated the exploration of alternative methods for tick control (Samish et al., 2004), such as the use of entomopathogenic fungi (EPF). Biological control by EPF is one of the most promising options for tick control (Polar et al., 2005). The most widely used EPF species against cattle ticks are *Metharizium anisopliae* s.l., *Beauveria bassiana* and *Akanthomyces lecanii* (formerly, *Lecanicillium lecanii*) (Fernandes et al., 2012; Romo-Martínez et al., 2013). EPF show clear advantages, such as being environmentally safe, can be mass-produced, and have the ability to infect their hosts through the cuticle rather than wait for ingestion in order to cause infection (Rajula et al., 2020). It has also been reported that EPF may affect the entire tick cycle (free-living and parasitic stages) (Fernández-Salas et al., 2017, 2018, 2019), a characteristic that allows broadening the spectrum of use in a tick control strategy. The research requires a transdisciplinary approach in order to be able to integrate the necessary knowledge on the use of EPF in the control of ticks. Through this integration, it will be possible to identify links between the studies carried out, generate research hypotheses to improve biological control and design viable EPF application schemes based on experiences that help guide future studies in the use of these fungi against livestock ticks. The objective of this paper is to review the use of EPF as an alternative control method against cattle ticks in Mexico.

### Cattle Ticks

**Figure 1:**
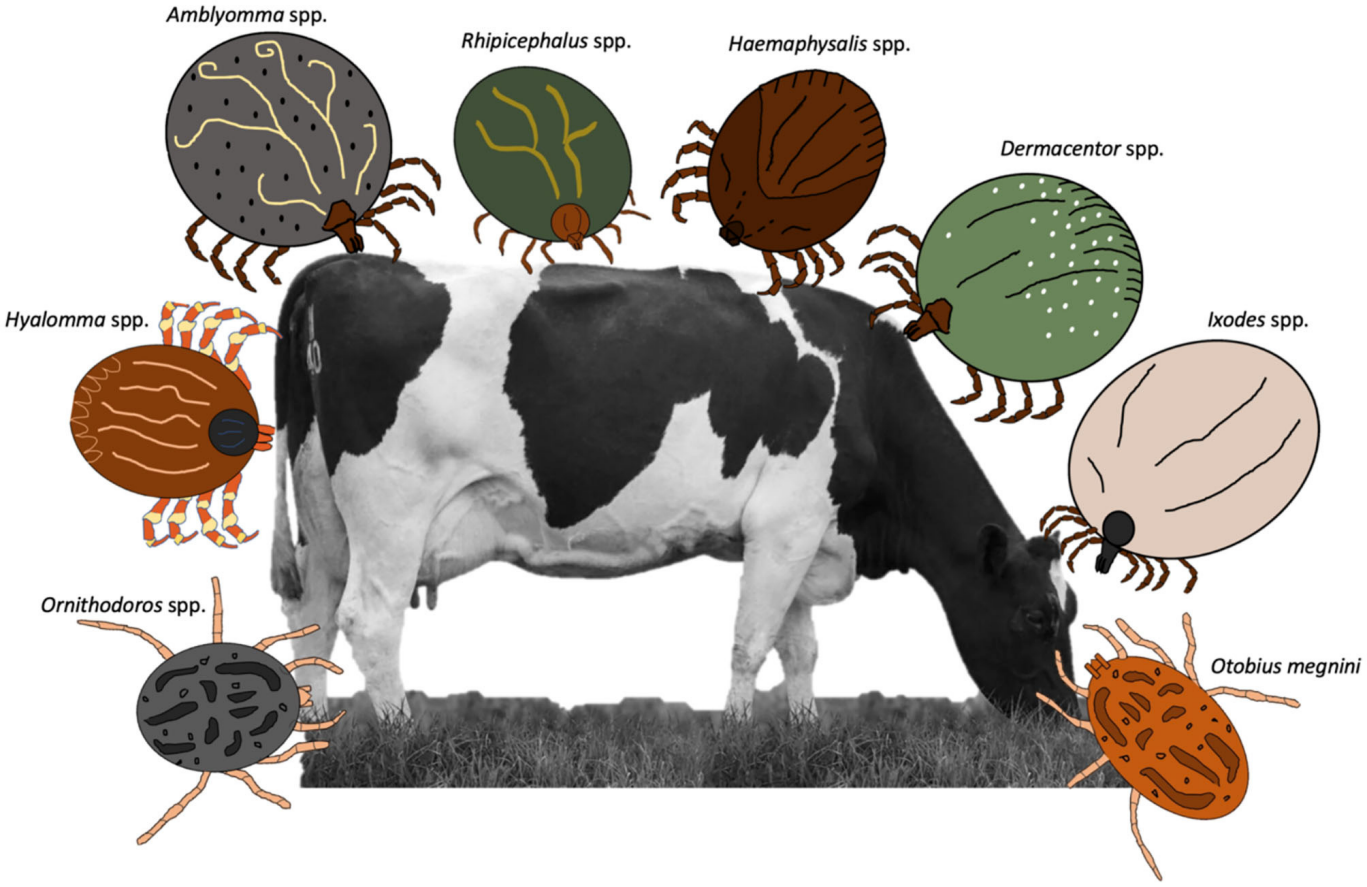
Ticks infesting cattle around the world.

Ticks are obligate blood-feeding ectoparasites that infest 80% of the cattle worldwide (Giles et al., 2014; Grisi et al., 2014). These ectoparasites are one of the most important health problems for the livestock industry and are responsible for high economic losses around the world, putting food safety at risk (Rodríguez-Vivas et al., 2017). In addition to having direct effects on their hosts, ticks are also the most important group of parasitic arthropods as vectors of pathogens that affect domestic animals and wildlife (Pérez de León et al., 2020). Tick-borne pathogens are the main cause of transboundary livestock diseases (e.g., bovine babesiosis, anaplasmosis, theileriosis, and heartwater disease), which are among the diseases listed as notifiable by the World Organization for Animal Health (Esteve-Gasent et al., 2020). Estimated annual global costs associated with ticks and the pathogens transmitted by them range between US$ 13.9 billion and US$ 18.7 billion (De Castro, 1997). Ticks that affect cattle around the world belong to two families: Ixodidae and Argasidae. The first, also known as hard ticks, includes all species from *Amblyomma, Dermacentor, Haemophysalis, Hyalomma, Ixodes*, and *Rhipicephalus*; while the second family or soft ticks, includes the *Ornithodoros* and *Otobius* ticks (Figure 1).

### Cattle Ticks in Mexico

In Mexico there are 82 species of ticks that parasitize domestic and wild animals (Higa et al., 2020). The main ticks of domesticated cattle belong to the Ixodidae family. Among these, *Rhipicephalus* (*Boophilus*) *microplus* (Canestrini), *R*. (*B*.) *annulatus* (Say), and *A. mixtum* (Koch 1844) have been reported with a high prevalence in cattle farms across the country. However, there are other ticks such as *Dermacentor albipictus, R*. *sanguineus, Anocentor nitens* and *Otobius megnini* that also have a considerable livestock impact (Martínez et al., 2019). In Mexico, the economic losses caused only by *R*. *microplus* were US$ 573.61 million per year (Rodríguez-Vivas et al., 2017). Although, some other ticks such as *Otobius megnini* which is present throughout the country, are also very important in livestock inspections for the export market (Martínez et al., 2019).

### Cattle Fever Ticks *Rhipicephalus microplus* and *Rhipicephalus annulatus*

**Figure 2:**
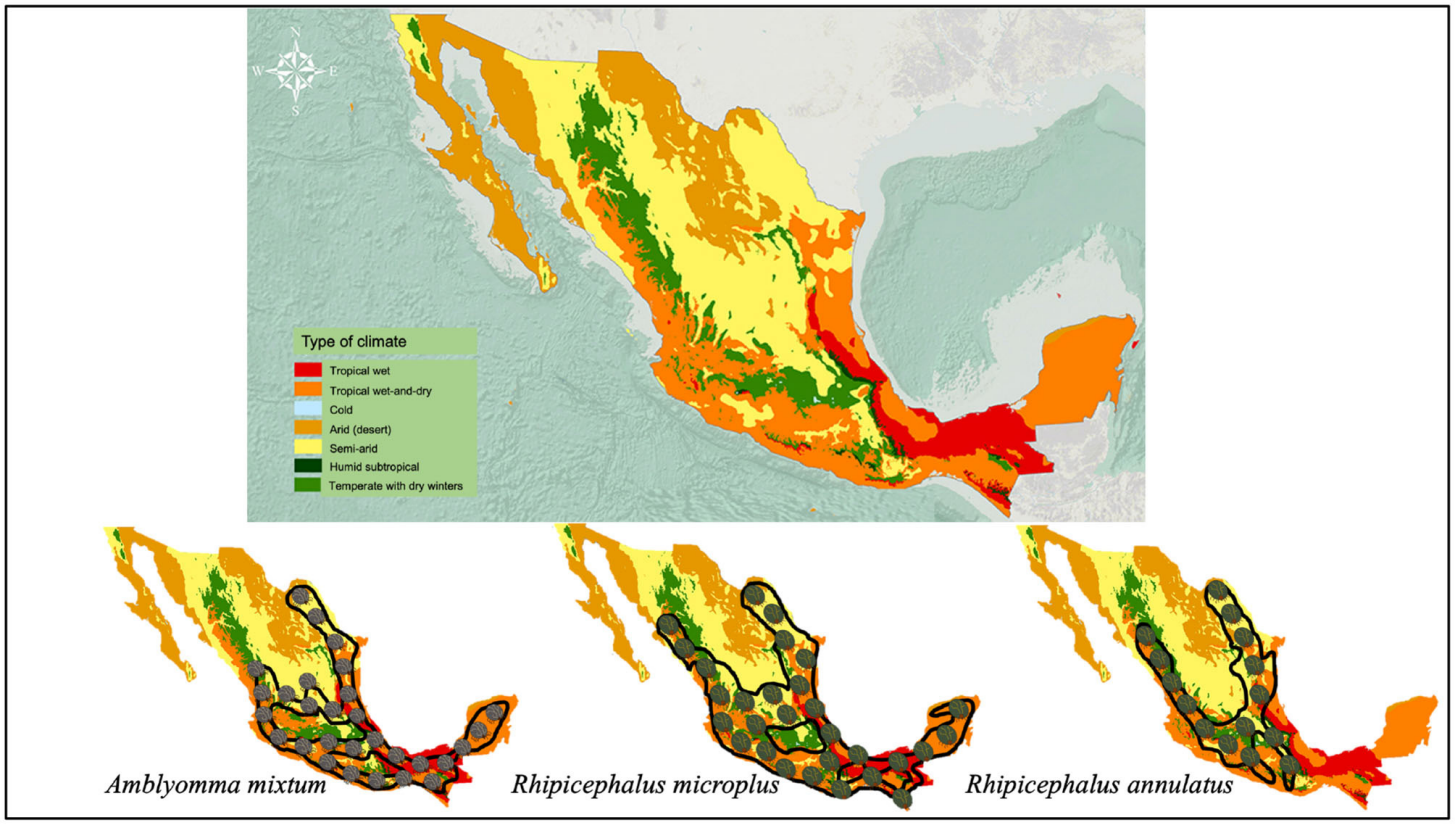
Climate zones of Mexico and their relationship with the distribution of the main ticks that affect cattle in Mexico. Imaged edited according to information from SEMARNAT (Secretaría de Medio Ambiente y Recursos Naturales) (2003) and SENASICA-SAGARPA (2015).

**Figure 3:**
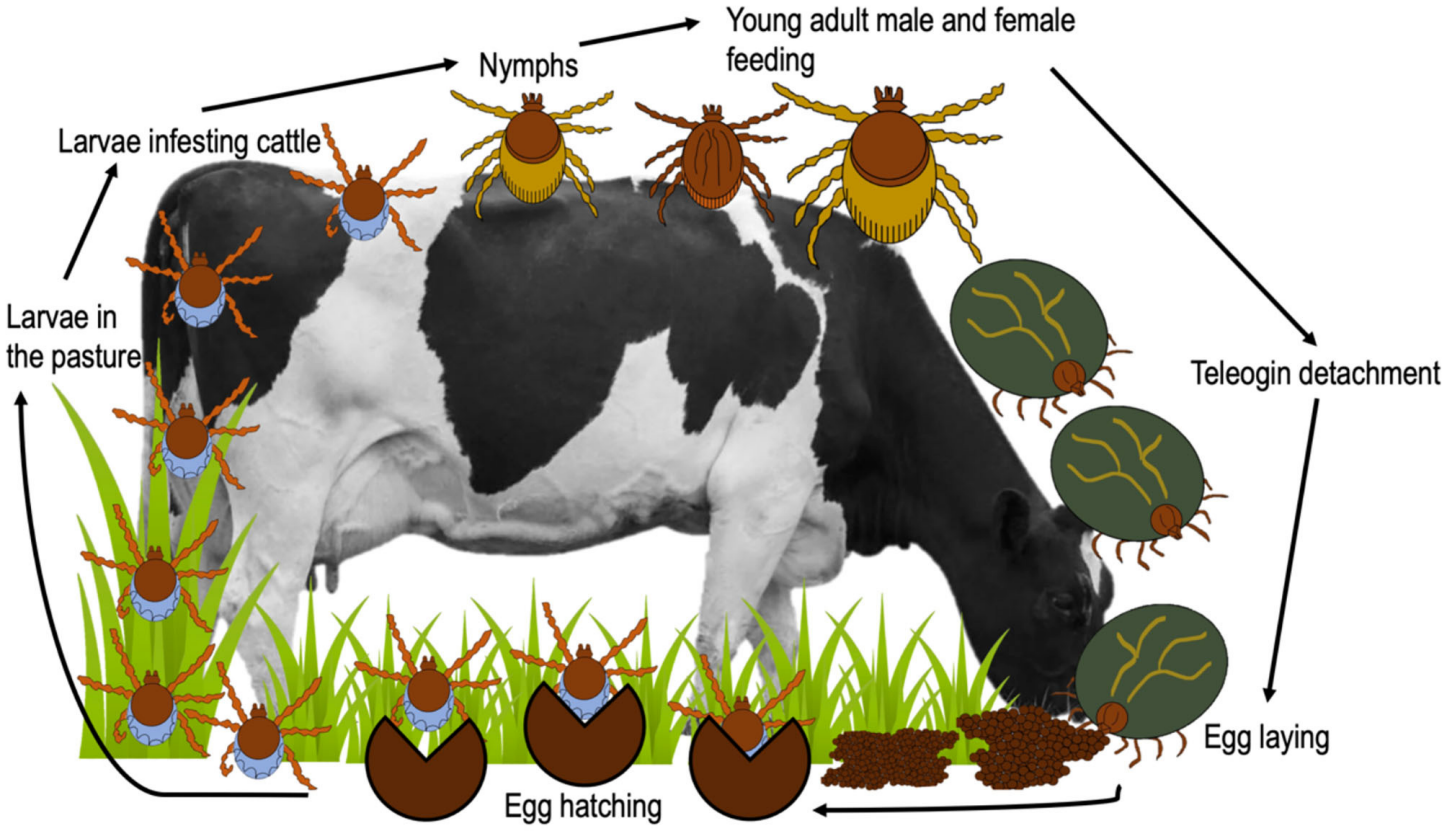
Life cycle of *R. microplus*.

Cattle fever ticks (CFT) *R*. *microplus* and *R*. *annulatus* remain endemic in Mexico (Esteve-Gasent et al., 2020). Both ticks have similar biological processes and morphology; however, their geographic distribution is different (Estrada-Peña and Venzal, 2006; SENASICA, 2013). While *R*. *microplus* is present in tropical and subtropical regions, *R*. *annulatus* is endemic to arid and semiarid regions (Northern Mexico) (SENASICA, 2013) (Figure 2). CFT are present in 65% of the national territory and have the capacity to infest mainly cattle, but they have also been reported to infest equines, deer and other wild animals (CFSPH (The Center For Food Security and Public Health), 2007; Rodríguez-Vivas et al., 2013a). These ticks have the ability to transmit notifiable animal diseases in cattle, such as anaplasmosis and babesiosis (Klafke et al., 2020). For this reason, CFT have special attention and constant vigilance in the border area between Mexico and the United States, in order to prevent their spread in free-tick areas (Lohmeyer et al., 2011), where there has already been an increase in infestations or outbreaks (Pound et al., 2010; Araya-Anchetta et al., 2015). The biological cycle of *R*. *microplus* is shown in Figure 3.

### Amblyomma mixtum

**Figure 4:**
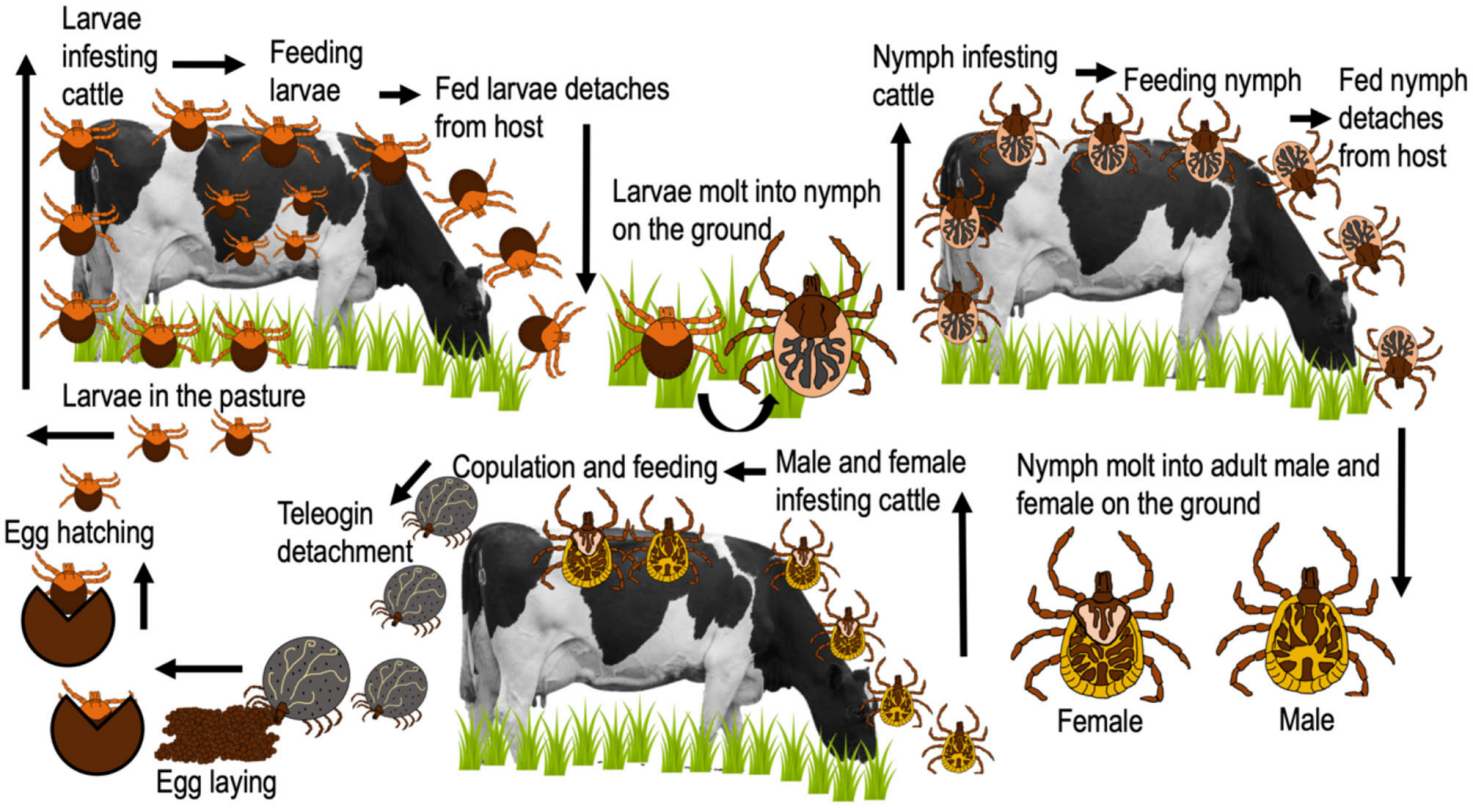
Life cycle of *A. mixtum*.

*A*. *mixtum* has a similar distribution to that of *R*. *microplus* in Mexico (Figure 2) (SENASICA, 2013); where, concomitant infestations are common in ~86% of farms (Alonso-Díaz et al., 2013a). Currently, it seems that *A*. *mixtum* has a greater distribution, since this species has been able to adapt to various ecological niches, including semi-arid grasslands and subtropical secondary forests (Estrada-Peña et al., 2004); in addition to its great capacity to occupy the ecological niches of other ticks (i.e., *R*. *microplus* under high pressure from acaricides) (Alonso-Díaz et al., 2013b). This ectoparasite has a heteroxenous life cycle and is a generalist species that infests livestock, humans and, wildlife in Mexico (Aguilar-Domínguez et al., 2019; Higa et al., 2020). It causes economic losses due to the large amount of blood taken from its hosts and the transmission of infectious diseases to domestic/wild animals (*Anaplasma marginale*) and humans (*Rickettsia ricketsii*) (Alonso-Díaz et al., 2013b; Aguilar-Domínguez et al., 2019). Additionally, other potentially zoonotic species such as *Rickettsia amblyommatis* have been detected in *A*. *mixtum* from Mexico (Sánchez-Montes et al., 2016; Merino et al., 2020), making this parasite one of the most important tick species in veterinary medicine and public health in the country (Pérez de León et al., 2020). The biological cycle of *A*. *mixtum* is shown in Figure 4.

Table 1 shows the temperature, rainfall and relative humidity per climate zone in Mexico to observe the characteristics of each ecological niche where the main ticks are distributed.

**Table 1:** Annual mean temperature, rainfall and relative humidity per climate zone in Mexico.

**Climatic zone**	**Mean temperature (^**°**^C)**	**Mean rainfall (mm)**	**Relative humidity (%) Min–Max**
Tropical wet	22–26	2000–4000	50.0–100
Tropical wet and dry	22–26 (>26 in some areas)	1000–2000	40.0–91.4
Cold	10–15	500–850	25.7–77.7
Arid (desert)	18–22	100–300	25.0–82.0
Semi-arid	18–26	300–600	25.7–77.7
Humid subtropical	18–22	2000–4000	40.0–91.4
Temperate with dry winters	10–22	600–1000	20.0–80.0

*Information obtaining from Prieto (2005) and Morillón et al. (2018)*.

### Impact of Climate Change on the Epidemiology of Ticks

Climate change is viewed as a long-term change in average weather patterns that have come to define Earth's local, regional and global climates (NASA-GCC, 2019). Perhaps, some of the most important alterations caused by climate change are warmer temperatures in temperate zones, altered precipitation patterns, increased frequency and severity of extreme weather events (hurricanes or droughts), and sea level rise (Kutz et al., 2009; Polley and Thompson, 2009). These last changes have affected, directly or indirectly, the biology and ecology of a great number of organisms on the planet; therefore, these climate variations have impacted on the habits and biological cycles of ectoparasites (Cumming and Van Vuuren, 2006; Kutz et al., 2009), including ticks (Pérez de León et al., 2012). In this regard, some authors in Mexico have mentioned that *R*. *microplus* can present between four to five successful generations per year in tropical and subtropical areas (Rodríguez-Vivas et al., 2005). Ticks have had the ability to evolve, adapt and spread within the changing climatic conditions, which, for the most part, have favored the dynamics and population movement of these arachnids in different geographical areas (Barré and Uilenberg, 2010). This situation has led to the presentation of relatively new infestations in some livestock areas, or the diagnosis of diseases transmitted by these vectors, which were not common for certain latitudes in the past (Estrada-Peña, 2008; Montero et al., 2016). Climate change can also affect domestic or wild hosts (Barré and Uilenberg, 2010; Rodríguez-Vivas et al., 2013a), which influences the geographical distribution of ticks, their infestations and the diseases they transmit in non-endemic areas (Giles et al., 2014). The presence of CFT has been frequently reported in tick-free zones or quarantine zones in the US. The risk of introducing ticks into or outside the quarantine zone is mainly high due to the movement of tick host species, such as the white-tailed deer (Pound et al., 2010; Webb et al., 2010), the nilgai antelope (Cárdenas-Canales et al., 2011), stray cattle and interactions between *R*. *microplus* and exotic weeds along the transboundary region with Mexico (Racelis et al., 2012; Esteve-Gassent et al., 2014). Likewise, the red deer (*Cervus elaphus*) has been reported as a wild host for the *R*. *microplus* tick (Rodríguez-Vivas et al., 2013a), helping it to spread within the Mexican territory. Obviously, the movement of these hosts is also closely related to human activities, the temperature increases in some areas, and the scarcity of water. All these characteristics can participate in a possible complex change in the ecology of ticks, since their biological cycles can be affected by these conditions.

### Tick Control

Tick control is mainly based on the use of chemical acaricides, which in recent decades have played a crucial role in the sustainability of the livestock production. However, since the development of the first broad-spectrum parasiticides, they have been used extensively by farmers in order to control or eliminate parasites. When ectoparasiticides are administered correctly (dosed and targeted), they are effective and have wide safety margins for both the animals and the people who apply them. However, there are factors such as resistant or multiresistant parasites and/or incorrect ways of applying the medications, which decrease their effectiveness (Alonso-Díaz et al., 2014). Currently, global results reveal that parasite control schemes based on a rigorous and exclusive use of chemical applications are not sustainable. The continuing propagation of these serious problems on a large scale involves many people in the pharmaceutical industries, professionals, farmers and in public health. It should be noted that chemical acaricides are and will be the fundamental basis of tick control, that is why they should be considered as a precious resource for cattle farming, since the cost of having an acaricide on the market implies expenses of more than 250 million dollars and between 8 and 12 years of research (De Alva, 1995; Omkar, 2016).

### Acaricide Resistance of Livestock Ticks in Mexico

**Figure 5:**
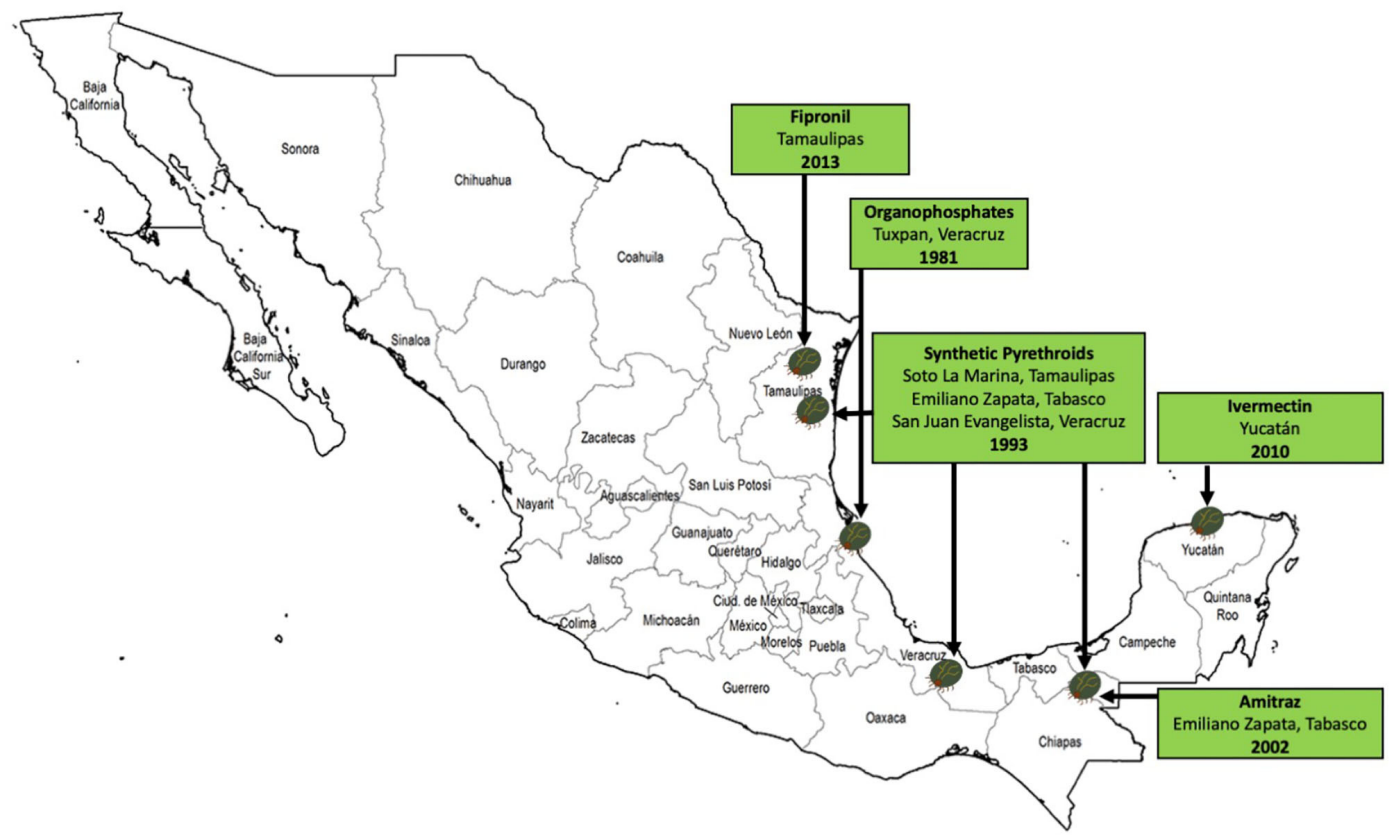
First reports of *R. microplus* tick populations resistant to acaricides in Mexico (Ortiz et al., 1995; Soberanes et al., 2002; Perez-Cogollo et al., 2010a; Miller et al., 2013).

One of the biggest concerns that has arisen on cattle farms across the country is the ability of ticks to resist the deadly effects of the chemicals used for their control. Tick resistance to acaricides is defined as “the specific heritable trait or traits in a tick population, selected as a result of the population's contact with an acaricide. This translates into a significant increase in the percentage of the population that survives after exposure to a certain concentration of this acaricide” (Rodriguez-Vivas et al., 2018). In Mexico, several investigations have been conducted to identify and monitor populations of resistant and multiresistant ticks to acaricides, and to know the risk factors associated with the presence of this growing problem (Fernández-Salas et al., 2012a,b,c; Alonso-Díaz et al., 2013a; Higa et al., 2020). Figure 5 shows the first cases of ticks resistant to acaricides in Mexico. Table 2 shows a summary of the epidemiological studies of resistant or multiresistant ticks by state over time in the country, highlighting *R*. *microplus*, which has developed resistance to all the main types of acaricides. Multiple acaricide resistance is an alarming phenomenon in Mexico, considering that there are no new synthetic compounds on the market with a novel mode of action to control multidrug resistant ticks (Esteve-Gasent et al., 2020).

**Table 2:** Main reports of resistance of *R*. *microplus* to acaricides by state in Mexico.

**State**	**Chemical Family**	**Acaricide/endectocide**	**References**
Yucatán	OP	Diazinon, Coumaphos, Chlorfenvinphos	Rodriguez-Vivas et al., 2006a
	SP	Flumethrin, Deltamethrin, Cypermethrin	Rodriguez-Vivas et al., 2006a, 2012; Rodríguez-Vivas et al., 2013b; Cabrera-Jimenez et al., 2008; Rosario-Cruz et al., 2009
	Am	Amitraz	Rodriguez-Vivas et al., 2006b; Rosado-Aguilar et al., 2008
	ML	Ivermectin	Perez-Cogollo et al., 2010a,b; Alegría-López et al., 2015
Veracruz	OP	Chlorpyrifos, Diazinon	Fernández-Salas et al., 2012c
	SP	Flumethrin, Deltamethrin, Cypermethrin	Fernández-Salas et al., 2012a,c
	Am	Amitraz	Fernández-Salas et al., 2012a
	ML	Ivermectin	Fernández-Salas et al., 2012b,c
Tamaulipas	OP	Diazinon, Coumaphos, Chlorfenvinphos, Lindane	Armendáriz-González, 2003
	SP	Flumethrin, Deltamethrin, Cypermethrin	
	PP	Fipronil	Miller et al., 2013
Tabasco	Am	Amitraz	Soberanes et al., 2002
Campeche	OP	Diazinon, Coumaphos	Li et al., 2003
	Am	Amitraz	Li et al., 2004
Nuevo León	OP	Diazinon	Miller et al., 2008
Coahuila	P	Permethrin	Miller et al., 2007
Chiapas	Am	Amitraz	Aguilar-Tipacamú et al., 2009

*OP, organophosphates; SP, synthetic pyrethroids; P, pyrethroids; Am, amidines; ML, macrocyclic lactones; PP, phenylpyrazoles*.

This type of resistance has been reported in different regions of Mexico and the most common in *R*. *microplus* are: coumaphos, flumethrin, and amitraz; chlorfenvinphos, flumethrin, and amitraz; diazinon, deltamethrin, and amitraz (Rodriguez-Vivas et al., 2007); permethrin, coumaphos, and fipronil; permethrin, coumaphos, fipronil, and amitraz (Miller et al., 2013); amitraz, cypermethrin, and ivermectin (Fernández-Salas et al., 2012a,b); and coumaphos, cypermethrin, amitraz, ivermectin and fipronil (Rodríguez-Vivas et al., 2014a). Although less studied, multiresistant strains of *A*. *mixtum* to acaricides have also been detected (Table 3). As for *R*. *annulatus*, there is insufficient evidence to know the resistance degree of this tick to chemical acaricides in Mexico; however, some studies suggest that it may be underdiagnosed as in some other countries (Klafke et al., 2020). Recently, the first evidence of permethrin resistance in *R*. *annulatus* strains was reported near the US-Mexico border, in Maverick County, Texas (Klafke et al., 2020). It is important to consider that populations of *R*. *annulatus* resistant to pyrethroids (Ziapour et al., 2017; Aboelhadid et al., 2018) and ivermectin have already been reported in other countries.

**Table 3:** Reports of *A*. *mixtum* resistant to acaricides in Mexico.

**State**	**Chemical family**	**Acaricide**	**References**
Veracruz	OP	Diazinon, Coumaphos, Chlorpyrifos	Alonso-Díaz et al., 2013a
	Am	Amitraz	Alonso-Díaz et al., 2013a; Higa et al., 2020

*OP, organophosphates; Am, amidines*.

Since acaricides will continue to be the basis of tick control, their lifespan and effectiveness need to be extended. To achieve this, it is suggested to know, evaluate and adopt other alternative control strategies in order to design an adequate integrated control scheme for ticks. It has been mentioned that the best way to control ticks in cattle farms is to combat them simultaneously in different ways (Alonso-Díaz et al., 2014; Pérez de León et al., 2020). By doing this, the parasites have less ability to defend themselves and develop resistance.

### Entomopathogenic Fungi

EPF are a species of fungal pathogens for arthropods (Rajula et al., 2020). They are considered cosmopolitan saprophytic organisms that live in diverse ecosystems and climates (e.g., tropical, temperate, arid and artic), where they interact with arthropods in many terrestrial and aquatic habitats (Skinner et al., 2014). It is estimated that there are between 750 and 1,000 EPF placed in more than 100 genera (Mantzoukas and Eliopoulos, 2020; Rajula et al., 2020), which play an important role in the dynamics of arthropod populations in natural ecosystems (Maina et al., 2018). EPF comprise a wide range of genera and species with high morphological, phylogenetic and ecological diversity (Araújo and Hughes, 2016), and their interactions with arthropods are of great interest for environmental microbiology, determination of the balance of ecosystems, biodiversity, evolution of eukaryotic organisms and insect pest control (Semenova et al., 2020). These fungi have the ability to evolve to be more successful in their biological cycle, and they are also capable of colonizing and using arthropods as a substrate for their reproduction (Humber, 2008). Among the arthropods to which they have adapted throughout this evolutionary transition are ticks, which is why they have been studied as a biological control for these pest arachnids. Biological control using EPF represents one of the most promising approaches for sustainable tick control schemes and can therefore be included as a component in an integrated pest management strategy for tick control. In addition, EPF have some advantages over conventional acaricides, such as: cost-benefit ratio, absence of harmful or secondary effects to non-target organisms, reduction of chemical residues in the environment and foods of animal origin, and short time between fungal generations (high production) (Porfirio and Schwentesius, 2016). On the other hand, EPF can protect biodiversity in the natural ecosystem and can be used in combination with synthetic chemical products, since their residues have no known adverse effects on the environment, and are self-perpetuating under ideal environmental conditions (Maina et al., 2018). For example, Webster et al. (2105) reported that the combination of *M*. *anisopliae* with commercial acaricides (cypermethrin and chlorpyriphos) enhance the tick control against *R*. *microplus* (97.9% of efficacy). EPF have also been shown to play additional roles in nature, including endophytism, antagonism of plant diseases, promoting plant growth, and rhizosphere colonization (Jaber and Ownley, 2018). The most studied EPF worldwide as biological control for ticks are *Metarhizium anisopliae* s.l., *B. bassiana* and *A. lecanii* (formerly, *Lecanicillium lecanii*) (Fernandes et al., 2012; Romo-Martínez et al., 2013). This is consistent with the reports that exist in Mexico (Ojeda-Chi et al., 2011; Fernández-Salas et al., 2018); however, some other EPF have been reported in the country, such as *Isaria* (*Paecilomyces*) *fumosorosea* (*fumosoroseus*), which has also stood out for its effectiveness (Ángel-Sahagún et al., 2010).

Currently, the taxonomic identification and reporting of *M*. *anisopliae* and *B*. *bassiana* strains are based on the studies proposed by Bischoff et al. (2009) and Rehner et al. (2011), respectively. These proposals are supported by various studies of molecular phylogeny of multiple loci and taxonomic classification, where various monophyletic lineages have been identified concluding that both species of fungi actually comprise a complex of species, which, in many cases, they are difficult to delimit without molecular tools and analysis. After these studies, where the taxonomy of the species is clarified, the strains of these EPF that have not been reidentified according to this current taxonomy should be reported as sensu lato (s.l.), and those that have been reidentified and delimited with the taxonomic techniques and proposed molecular phylogenetic studies will be reported as sensu stricto (s. str). The EPF strains used in Mexico for the control of cattle ticks have been identified through morphological analysis of their reproductive structures, and some through molecular analysis. However, in the case of *M*. *anisopliae* and *B*. *bassiana*, some of the molecular identification techniques used were not sufficient for the delimitation of the monophyletic lineages and, for other strains, the information from the molecular analyzes is not available. Therefore, in the present review, the strains of these fungi used in Mexico will be considered as *M*. *anisopliae* sensu lato (s.l.) and *B*. *bassiana* sensu lato (s.l.) as well.

### Infection Mechanism of EPF

**Figure 6:**
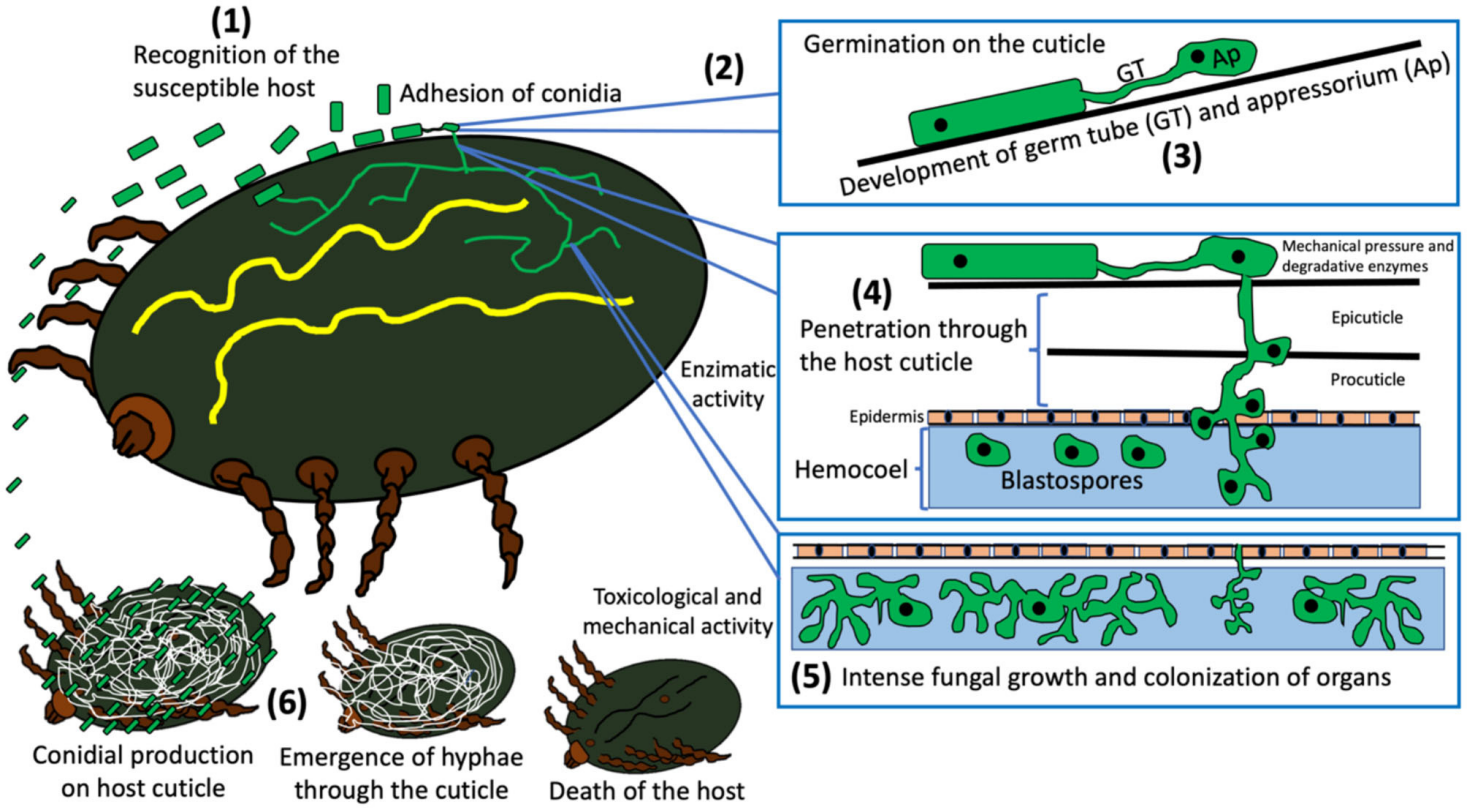
General infection process of entomopathogenic fungi in ticks and their biological mechanisms.

The basic advantages related to the infection mode of EPF, compared to commercial acaricides, correspond to their ability to use different mechanisms to colonize and kill ticks. Fungi use enzymatic, toxicological and mechanical invasion systems, which suggest a difficulty for ticks to develop resistance to EPF. Furthermore, it is known that they can target almost all stages of the arthropod life cycle, which means another great advantage as a member of pest control schemes (Srinivasan et al., 2019). According to Beys-da-Silva et al. (2020), the infection mode of EPF in ticks occurs as follows: (1) recognition of the susceptible host; (2) adhesion of conidia and germination on host cuticle; (3) development of specific structures (germ tube and appressorium); (4) penetration through the host's cuticle; (5) intense fungal growth and death of the host; and (6) production of conidia after hyphae emergence through the host cuticle. Figure 6 schematizes the infection mode of EPF in ticks.

### Recognition of Susceptible Host, Adhesion of Conidia and Germination on the Host Cuticle

Aerial fungal conidia adhere to the host's cuticle through hydrophobic mechanisms (Ortiz-Urquiza and Keyhani, 2013), which are mainly mediated by proteins on the surface of the conidia named hydrophobins (St. Leger et al., 1992; Skinner et al., 2014) and adhesins (i.e., Mad1 and Mad2 identified in *M*. *anisopliae*) (Wang and St Leger, 2007; Valero-Jiménez et al., 2016). It is important to mention that homologous proteins of these adhesins have been reported in *B*. *bassiana* (Gao et al., 2020), but it has been suggested that some genes that encode these proteins between the two main EPF (*M*. *anisopliae* and *B*. *bassiana*) could be different (Chen et al., 2018). Beys da Silva et al. (2010a) reported that lipolytic activity due to the action of enzymes such as lipase and esterase in ticks, could also contribute to the recognition and adhesion of conidia during the infection of *R*. *microplus* by *M*. *anisopliae*.

### Penetration Through the Host's Cuticle

Once the conidia are attached, they will germinate under optimal humidity and temperature conditions, producing a germination tube followed by a peg or appressorium for penetration into the host's cuticle (Skinner et al., 2014; Brunner-Mendoza et al., 2019). The penetration process begins and is aided by the production of several hydrolytic cuticular enzymes such as lipases, proteases and chitinases, and the mechanical pressure exerted by the appressorium (Brunner-Mendoza et al., 2019). Lipolytic enzymes including lipases, act primarily on the epicuticle, followed by proteases and chitinases, according to the presence of polymeric substrates in the different portions of the cuticle (Beys da Silva et al., 2010a,b). Among the proteases that can act at this level are subtilisins, trypsins, chymotrypsins, metallopeptidases, aspartyl peptidases, and exopeptidases (Semenova et al., 2020); where their expression from fungi such as *M*. *anisopliae* will depend specifically on the composition of the cuticle and hemolymph (Freimoser et al., 2005). EPF such as *M*. *anisopliae* and *B*. *bassiana* can express up to 11 different subtilisins, one of the most important being the Pr1 subtilisin-like peptidases, which intervene in the arthropod pathogenesis, causing hydrolysis of the cuticle and providing nutrients to the fungus (Gao et al., 2020; Semenova et al., 2020). To our knowledge, there are few reports elucidating the participation of these proteases during the infection process of cattle ticks (*R*. *microplus, R*. *annulatus* and *A*. *mixtum*) by EPF. In this regard, Golo et al. (2015) reported that spores of *M*. *anisopliae* s.l. expressed the Pr1 gene and that there was an increase in the specific activity of Pr1, when the fungus was cultured in *R*. *microplus* larvae; however, these authors concluded that increased Pr1 activity in conidia and its expression levels were not associated with significant changes (up or down) in larval mortality. It was recently reported that five of the 11 members of the Pr1 family are essential for the maintenance of the total extracellular activity of Pr1, which is necessary for the degradation of the host cuticle during hyphal invasion of EPF (such as *B*. *bassiana*), providing capacity for a broadest host spectrum (Gao et al., 2020). We suggest that it is necessary to continue evaluating the participation of subtilisins produced by EPF during the infection process against cattle ticks. A full understanding of the pathogenicity and/or virulence mechanism is essential for the development of an effective biological control scheme.

### Fungal Growth and Death of the Host

After penetration and once inside the host, the EPF develop hyphal bodies and blastopores that multiply and disseminate through the hemolymph to invade different tissues (Maina et al., 2018; Beys-da-Silva et al., 2020) using circulation as a vehicle for colonization and for nutrient absorption (Valero-Jiménez et al., 2016; Brunner-Mendoza et al., 2019). During this event, different virulence factors act on host colonization in order to spread inside the arthropod's body, causing its death. Among the most important factors are mycotoxins such as Beauvericin, Beauverolides, Bassiannolide (by *B*. *bassiana, V*. *lecanii*, and *Paecilomyces* spp.) and Dextruxins A, B, C, D, E, F (by *M*. *anisopliae*), which act as poisons for the host (Maina et al., 2018). These fungal toxins might cause flaccid paralysis, cellular alterations and inhibit the normal functioning of muscle tissues, the middle intestine and the Malpighian tubes (Mora et al., 2017). After the dead of the host and when the nutrients within it are depleted, the fungus open the integument, forms aerial mycelia and carries out sporulation on the corpse, initiating the dispersal of its conidia (Valero-Jiménez et al., 2016). As we can see, EPF can have a wide variety of toxins that could affect the biology of livestock ticks. Although the general mechanisms of infection have been described for most of the useful EPF against arthropods, including ticks, the scientific community needs to understand the complexity of the molecular mechanisms in each infection phase, which is not completely elucidated. A transdisciplinary approach is required to incorporate different tools, such as genomics, transcriptomics, proteomics and metabolomics in order to better comprehend the mechanism of pathogenicity in EPF against ticks. It is important to note that not all EPF populations have the same capacity to produce all the enzymes or toxins reported in the scientific literature (Schrank and Vainstein, 2010), neither in variety nor in quantity. For example, in *R*. *microplus*, the immersion or inoculation of dextruxin A from *M*. *anisopliae* on engorged ticks neither affected their biological parameters nor caused paralysis (Golo et al., 2011); but other study reported an acaricidal effect of distinct destruxins from Beauveria feline against *R*. *microplus* (Morais-Urano et al., 2012). In addition, different environmental and nutritional factors have been identified as the main triggers of the genetic expression of these components (Campos et al., 2005; Fang et al., 2005). The production capacity of these toxins and enzymes is reflected in the time of death of the tick, which varies according to the EPF strain, the type of fungus (genus and species), the treated tick genus, the method of application and the number of infectious spores (Fernández-Salas et al., 2017; Mantzoukas and Eliopoulos, 2020). Pathogenicity or virulence factors give EPF the ability to be specific to one or other pest arthropod, a characteristic that helps control them, without affecting other organisms beneficial to nature (Kirkland et al., 2004). It should be noted that EPF are considered “non-specialized” mite pathogens, and it has not been conclusively demonstrated that they develop specificity against ticks to the extent of causing epizootics (Fernandes et al., 2012), as has happened with some insects such as *Lymantria dispar* (Lepidoptera: Erebidae), *Diprion pini* (Hymenoptera: Diprionidae), *Dendrolimus pini* (Lepidoptera: Lasiocampidae), *D. punctatus* (Lepidoptera: Lasiocampidae), *Malacosoma disstria* (Lepidoptera: Lasiocampidae), *Fiorinia externa* (Hemiptera: Diaspididae) (Augustyniuk-Kram and Kram, 2012). However, several authors have mentioned the high capacity of these organisms to become specialists for certain arthropods, without losing their ability to be general pathogens (Beys-da-Silva et al., 2020). In this regard, a recent study has found an increase in mortality caused by Mexican strains of *M*. *anisopliae* s.l. on *R*. *microplus* and *A*. *mixtum*, after submitting them to four passages using these ticks as substrate (Romero-Pérez, 2020). Furthermore, Adames et al. (2011) reported in Mexico that four to seven passages of *M*. *anisopliae* s.l. on *R*. *microplus* increase its virulence against this tick. Although more studies are needed in this regard (e.g., what are the molecular or metabolic mechanisms that are triggered to increase this virulence?), the mortality behavior that these EPF develop in their evaluation against ticks is interesting. This possibility shows a promising outlook to maintain or improve virulence in those Mexican EPF strains that show favorable characteristics such as high sporulation, resistance or tolerance to UV rays, thermotolerance and/or probable specificity against ticks.

## Entomopathogenic Fungi as Livestock Tick Pathogens

### Laboratory Tests

The evaluations of *in vitro* studies regarding the acaricidal effect of EPF against livestock ticks (including resistant populations) and their reproduction in Mexico are shown in Tables 4–6. Most laboratory studies have demonstrated the effectiveness of EPF and, in some cases, their potential to control susceptible and resistant/multiresistant ticks; however, the following details can also be observed and summarized.

**Table 4:** Laboratory evaluations of the EPF effectiveness (dosage: 1 × 10^8^ conidia/ml) against engorged female of *R*. *microplus* (including resistant populations) in Mexico.

**EPF**	**Strain**	**Mortality %(evaluation days)**	**References**
*M*. *anisopliae*	ESC1	100 (20)^*^	Fernández-Ruvalcaba et al., 2005
	M379	37.78–53.33 (15)**	Adames et al., 2011
	Ma14	100 (20)	Ojeda-Chi et al., 2010
	Ma34	100 (20)	Ojeda-Chi et al., 2010
	Ma14+Ma34	100 (20)	Ojeda-Chi et al., 2010
	5 strains	87–100 (20)	Alcalá-Gómez et al., 2017
	55 strains	3.3–100 (20)^***^	Fernández-Salas et al., 2017
*B*. *bassiana*	3 strains	84–100 (20)	Alcalá-Gómez et al., 2017
	6 strains	3.3–86.7 (20)^***^	Fernández-Salas et al., 2018

* *Including populations resistant to OP (organophosphates);*

*** *including populations resistant to OP and SP (synthetic pyrethroids);*

***including populations resistant to OP, SP, Am (amidines) and Iv (ivermectin).

**Table 5:** Laboratory evaluations of the EPF effectiveness (dosage: 1 × 10^8^ conidia/ml) against larvae of *R*. *microplus* and *A*. *mixtum* (including resistant populations) in Mexico.

**EPF**	**Strain**	**Ticks**	**Mortality %(evaluation days)**	**References**
*M*. *anisopliae*	33 strains	*R*. *microplus*	2–100 (NS)	Ángel-Sahagún et al., 2010
	Ma14	*R*. *microplus*	45–62 (20)	Ojeda-Chi et al., 2010
	Ma34	*R*. *microplus*	34–57 (20)	Ojeda-Chi et al., 2010
	Ma14+Ma34	*R*. *microplus*	90 (20)	Ojeda-Chi et al., 2010
	5 strains	*R*. *microplus*	64.6–100 (16)	Cruz-Avalos et al., 2015
	3 strains	*R*. *microplus*	69.2–78.5 (4)	Díaz et al., 2014
	54 strains	*R*. *microplus*	1.7–100 (20)^*^	Fernández-Salas et al., 2019
*B*. *bassiana*	4 strains	*R*. *microplus*	2.5–42.9 (16)	Cruz-Avalos et al., 2015
	6 strains	*R*. *microplus*	3.2–53.2 (20)^*^	Fernández-Salas et al., 2019
*I*. *fumosorosea*	20 strains	*R*. *microplus*	5–94 (NS)	Ángel-Sahagún et al., 2010
	Ifr22	*R*. *microplus*	28.6 (16)	Cruz-Avalos et al., 2015
*P. lilacinum*	PlV1	*R*. *microplus*	92.3–94.9 (20)^*^	Fernández-Salas et al., 2019
*M. anisopliae*	23 strains	*A*. *mixtum*	0–32.7 (20)	Jiménez-Ruíz, 2015
*B. bassiana*	2 strains	*A*. *mixtum*	0–1.9 (20)	Jiménez-Ruíz, 2015

* *Including populations resistant to OP, SP, Am and Iv; NS, Not specified*.

**Table 6:** Effect of EPF at laboratory level (dosage 1 × 10^8^ conidia/ml) on the reproductive parameters of *R*. *microplus* in Mexico.

**EPF**	**Strain**	**Inhibition of oviposition %(evaluation days)**	**Inhibition of egg hatching (%)**	**References**
*M*. *anisopliae*	ESC1	74.6–75.2 (10)	Without effect	Fernández-Ruvalcaba et al., 2005
	M379	72.48–83.94 (15)	Not evaluated	Adames et al., 2011
	Ma14	12.5 (10)	Not evaluated	Ojeda-Chi et al., 2010
	Ma34	55.5 (10)	Not evaluated	Ojeda-Chi et al., 2010
	Ma14 + Ma34	39.1 (10)	Not evaluated	Ojeda-Chi et al., 2010
	5 strains	14–73 (20)	20–86	Alcalá-Gómez et al., 2017
	55 strains	8.24–55.68 (12)	Without effect	Fernández-Salas et al., 2017
*B*. *bassiana*	6 strains	0–38.2 (12)	Without effect	Fernández-Salas et al., 2018
	3 strains	12.4–98 (20)	36.7–98	Alcalá-Gómez et al., 2017

#### Most Studies Have Been Using *R. microplus* as a Model

There is only one report on the acaricidal effect of EPF against *A*. *mixtum* larvae and to our knowledge, there are not reports on the acaricidal effect on *R*. *annulatus* in Mexico. *R*. *microplus, R*. *annulatus* and *A*. *mixtum* are the most prevalent ticks on cattle farms across the country, causing great economic losses. Therefore, it is important to evaluate the acaricidal effect of fungi on a higher diversity of ticks in order to identify strains that have a wide or reduced action spectrum, since this information could help design tick control schemes in the field (i.e., 86% of cattle farms have cohabiting *R*. *microplus* and *A*. *mixtum* in Mexico), explore the EPF infection mechanisms in each tick genus, and elucidate some biological aspects of the fungal-host immune system interaction. In recent studies, several strains of *M*. *anisopliae* s.l. were highly effective against larvae of *R*. *microplus*, but not against *A*. *mixtum* larvae (Jiménez-Ruíz, 2015; Fernández-Salas et al., 2017). In other countries, a high variability in the virulence of EPF against different genera of ticks and different tick populations of the same genus has been reported (Fernandes et al., 2012; Perinotto et al., 2012). Webster et al. (2017) also reported that field populations of *R*. *microplus* show high variation in their susceptibility to *M*. *anisopliae*. It is necessary to understand the factors that are involved in this differentiated response, and especially to be able to find a strain of EPF with a broad acaricidal effect against different genera of ticks and different field populations.

#### Acaricidal Activity of EPF Against Resistant and Multiresistant Populations of *R. microplus*

An essential goal of implementing EPF in tick control programs is the mitigation of the economic and sanitary impact of the resistance that these arthropods have developed. When a tick population is resistant to chemical acaricides, it is inappropriate to neglect the possible interference of tick resistance mechanisms in the EPF performance (Perinotto et al., 2012). In Mexico, several strains of *M*. *anisopliae* s.l. induced a mortality of 90–100% in populations of *R*. *microplus* multiresistant to acaricides (OP, SP, Am) and ivermectin (Fernández-Salas et al., 2017, 2018, 2019). In fact, some studies reported a higher susceptibility to the EPF effect in *R*. *microplus* resistant to OP and SP than in susceptible strains (Adames et al., 2011). Fernández-Ruvalcaba et al. (2005) reported a similar mortality caused by *M*. *anisopliae* s.l. in *R*. *microplus* populations susceptible and resistant to OP. In general, *M*. *anisopliae* strains showed high acaricidal effects against resistant or acaricide-susceptible *R*. *microplus* and no differences in effects were observed between tick populations (Table 4). This is important because the resistance mechanism used by ticks in order to avoid the toxic effects of chemical acaricides did not affect the action mechanism of the EPF strains. This supports the opinion of some authors, who state that resistance to biological agents as EPF is less likely to occur compared to resistance to chemical acaricides (Polar et al., 2005).

#### Most Studies Have Been Targeting on a Single Stage of the Tick Life Cycle

A great advantage of EPF, compared to chemical acaricides, is that they can attack almost all stages of the arthropod life cycle, making them a unique component in integrated pest management approaches (Rajula et al., 2020). The few reports that exist on *A*. *mixtum* in Mexico were made in larvae, but the effect of EPF on adult ticks remains unknown. Regarding *R*. *microplus*, only a few EPF strains have been evaluated in engorged adult ticks and in larvae. A greater susceptibility of tick larvae to the lethal effect of EPF has been reported compared to engorged females (Fernandes and Bittencourt, 2008; Fernandes et al., 2012); however, it seems that in the bioassays carried out in Mexico there is a possible tendency of susceptibility in engorged adult ticks than in the larval stage. It would be interesting to evaluate the factors that could intervene within this possible tendency with Mexican EPF strains, considering different factors such as the nutritional, genetic, origin and biological conditions of both fungi and ticks. Regarding tick reproduction, EPF have shown a significant inhibition of oviposition (most strains cause more than 50% effectiveness in a short time) (Table 6). However, the fungal effect on egg hatching inhibition percentages is not reported or has not been evaluated. This could be different in the field, since in the laboratory, the egg mass is generally removed from the engorged female between 10 to 15 days from the beginning of oviposition in order to evaluate hatching, which avoids future contact of these eggs with the spores produced on the surface of teleogin corpses, a situation that would not occur in the field. Therefore, it is recommended to include in the evaluations the direct application of EPF in the egg mass to determine its effect on its viability. In addition, it is also advisable to carry out simultaneous studies that include all stages of the tick life cycle.

#### Most Studies Have Been Based in the Use of *M. anisopliae* s.l. and *B. bassiana* s.l. Strains

Most of the studies carried out in Mexico have used *M*. *anisopliae* s.l. as the main EPF against ticks and, to a lesser extent, *B*. *bassiana* s.l. and *I*. *fumosorosea*. However, according to genetic diversity studies, other fungi have been identified that could cause high mortality effects in ticks, such as *Purpureocillium lilacinum* (Fernández-Salas et al., 2019). Therefore, it is highly recommended to continue research that identifies and evaluates the effect of different genera and species of EPF against ticks in livestock. Also, it is important to highlight that the main states where these fungi have been isolated and evaluated are located in the tropical and subtropical areas of Mexico, which stand out for their extensive livestock activity and reports of tick resistance to conventional chemical acaricides. On the other hand, these studies have allowed not only to have different EPF strains effective against ticks, but also to isolate strains from different sources and the standardization of techniques through various bioassays in distinct laboratories in the country. Taking all these aspects into account for future research, it will help to improve the EPF's effect against ticks, and include them as an important tool in tick control programs. It should be noted that the main EPF used in the studies have been isolated from different orders of insects, ticks and culture soils. In recent studies, EPF isolates have been made directly from grassland soils (Bautista-Gálvez et al., 2017; Fernandez-Salas et al., 2020), investigating whether this native characteristic may influence the tickcide effect. So far, the results have been promising (Fernández-Salas et al., 2017, 2019), attributing them to coexistence with ticks and the evolutionary adaptation of EPF to various nutritional substrates. In Mexico, studies in which EPF are isolated directly from tick corpses are scarce, although it would be interesting to carry out investigations in search of naturally colonized ticks to determine their virulence and effectiveness under controlled conditions.

### Field Tests

Field studies evaluating the acaricidal effect of EPF against livestock ticks in Mexico are presented in Table 7. These studies have shown satisfactory control efficiency of *M*. *anisopliae* s.l. against different stages of *R*. *microplus* when applied both in pastures as in cattle; however, the following details can also be observed and summarized.

**Table 7:** Field evaluations (on cattle and pastures) of the EPF effectiveness against various biological stages of *R*. *microplus* ticks in Mexico.

**EPF**	**Strain**	**Biological cycle stage**	**Control %(evaluation days)**	**References**
*M*. *anisopliae*	Ma14	Larvae on pastures	58.3–94.2 (14–28)	Ángel-Sahagún et al., 2010
	Ma14+Ma34	Larvae on pastures	40.3–100 (28–35)	Ojeda-Chi et al., 2010
	Ma379	Larvae on cattle	99.5 (14)	Romo-Martínez et al., 2013
	Ma379	Nymphs on cattle	99.7 (14)	Romo-Martínez et al., 2013
	Ma14+Ma34	Larvae and nymphs on cattle	36.5–72 (until from 4 treatment) (42)	Rodríguez-Alcocer et al., 2014
	Ma34	Adults on cattle	45.7 – 91.2 (until from 4 treatment) (42)	Alonso-Díaz et al., 2007
	Ma198	All stages on cattle	88.5 (7)	Rivera-Cervantes et al., 2017
	Ma379	Adults and teleogins on cattle	95.4–98.17 (14)	Romo-Martínez et al., 2013
	MM01	Adults on cattle	47.7 (10) 37.7 (44.5)	Bautista-Gálvez et al., 2017
	Ma14+Ma34	Adults on cattle	60.6–84.0 (until from 4 treatment) (42)	Rodríguez-Alcocer et al., 2014
*B*. *bassiana*	*B*. *bassiana*	Adults on cattle	76.6 (37.3)	Bautista-Gálvez et al., 2017

#### Some Field Studies Have Demonstrated the Effectiveness of *M. anisopliae* on *R. microplus* Free-Living Stage (Larvae)

Worldwide, tick control has been based on therapeutic treatments mainly directed at parasitic stages of *R*. *microplus*. However, this tick spends between 80 and 90% of its life cycle outside the host in pastures (Needham and Teel, 1991); therefore, a good strategy to reduce the negative effects of tick infestations on cattle is to reduce free-living populations. *M*. *anisopliae* conidia can be applied to livestock farm pastures (as in crops) to control free-living stages. In Mexico, the aerial dispersal of *M*. *anisopliae* conidia in pastures reduced the number of *R*. *microplus* larvae from 40 to 100% (Ángel-Sahagún et al., 2010; Ojeda-Chi et al., 2010). However, it is necessary to investigate the acaricidal effect using another genus of EPF (e.g., *B*. *bassiana, I. fumosorosea* or *P. lilacinum*). Recently, our research group evaluated the acaricidal activity of about 60 EPF strains isolated from paddocks against *R*. *microplus* in the Mexican tropics (Fernández-Salas et al., 2017, 2018). EPF adapted both to the regional environmental conditions in which they were used, as well to the ticks that served as substrate for fungi development. Therefore, these EPF strains could also be useful in the implementation of biological control programs against ticks. In addition, several isolates showed thermotolerance and resistance to UV-R, which makes them possible candidates for field evaluation. Likewise, it is necessary to evaluate the fungal strains with high efficiency against other genera of ticks (*A*. *mixtum* and *R*. *annulatus*) present in pastures of cattle farms in Mexico.

#### Most *in vivo* Studies Have Demonstrated the Efficacy of *M. anisopliae* s.l. Applied to Naturally Infested Cattle

Most of the field studies carried out in Mexico showed that when EPF conidia were sprayed on cattle, high control percentages against parasitic stages of *R*. *microplus* were observed (Table 7). However, it is known that EPF could decrease its acaricidal efficacy against ticks under field conditions due to biotic and abiotic factors that affect its virulence and pathogenicity. Fungal growth, conidia production, survival, germination, pathogenicity, virulence and the production of bioactive compounds can be strongly influenced by exposure to ultraviolet solar radiation (Wong et al., 2019).

Therefore, it is recommended that when using EPF for tick control in the field, strategies to counteract the negative effects of these factors need to be considered in order to improve the tickcide effect. Among the main strategies are the use of protectors and dispersers of conidia from highly virulent and pathogenic isolates, the selection of isolates adapted to the climatic conditions where they will be used (i.e., native isolates with a greater natural tolerance to UV-R) and proper application of EPF in order to avoid high temperatures and UV-R.

#### No Adverse Effects Were Reported in Animals and/or Operators

An important point that should be taken into account when using EPF is the safety in their use. None of the field studies conducted has reported adverse reactions in cattle or operators. This is consistent with Zimmermann (2007), who mentioned that EPF do not pose risks to animals, humans or the environment, concluding that this control method is a safe and sustainable alternative. On the contrary, the use of chemical acaricides can be highly harmful to beneficial species or non-target organisms, humans, animals and the environment (Fernández-Salas et al., 2012a). Therefore, having an alternative control will help reduce the use of these chemicals and keep these situations at a low risk of presentation, based on the premise that EPF with affinity for a target organism are less capable of causing harm to non-target organisms (Goettel and Johnson, 1992). However, in Mexico, it is recommended to complement field studies with evaluations of the beneficial or negative impact of EPF on the ecosystems of livestock farms.

### Integrated Pest Management of Livestock Ticks

Through many years of experience in treating ticks, studies have concluded that applying a single treatment will not maintain efficient and sustainable control in the long term. Invariably, the product used will exhibit inefficiencies in killing ticks due to their ability to become resistant. In Mexico, it has been mentioned that none of the previous strategies (chemical and non-chemical) by themselves have been sufficient to sustainably control ticks, such as *R*. *microplus* (Romo-Martínez et al., 2013; Fernández-Salas et al., 2019). Therefore, it is necessary to integrate two or more methodologies in order to be able to attack ticks on several fronts and take care, among themselves, of the effectiveness of the products or techniques used. Integrated pest management is defined as the systematic application of two or more technologies that are compatible with each other, with the environment and that are profitable to control populations of arthropod pests that negatively affect livestock (Bram, 1994). EPF are compatible with various tick control products, including chemical acaricides, without losing their acaricidal capacity (Sousa et al., 2011; Kiss et al., 2012; Romo-Martínez et al., 2013). Therefore, the integration of EPF in a tick control scheme is totally feasible.

### Proposals for Integrated Tick Control in Mexico

Research evaluating integrated pest management schemes, including the EPF for tick control in Mexico, is scarce. There is a very important gap that requires more research, since it has been mentioned that integrated tick control is the best way to establish sustainable and successful livestock in order to increase the productive capacity of the animals (Rodríguez-Vivas et al., 2014b).

Proposals for the use of EPF within an integrated tick pest management should be designed according to several factors, listed below.

#### The Climatological Characteristics of the Region Where the Control Will Be Implemented

The population dynamics of ticks depends mainly on the conditions of temperature, relative humidity and rainfall. Therefore, the distribution of ticks throughout the year can be predicted since climatic factors are responsible for this characteristic.

#### The Ticks Present in the Control Area

Different tick genera may show differences in the biological cycles, so their presence in the bovine body and in the pastures is distinct throughout the year (e.g., *R*. *microplus* and *A*. *mixtum*, which coexist in the Mexican tropics).

#### The Toxicological Response/Behavior of Ticks

It is necessary to know the susceptibility status of the ticks to be treated, since, as mentioned above, chemical acaricides are and will be the basis of tick control programs, including those of integral management and the success of the establishment of these protocols will depend on their proper use.

#### The Availability and Compatibility of Various Methods for Tick Control

It is important to consider all available and proven alternatives for tick treatment and use them in combination with each other and with chemical acaricides.

One of the main advantages that is present in Mexico for the design of tick control protocols through integrated management is that climatic characteristics of the country have well-defined patterns (Estrada-Peña et al., 2006). However, there are few studies where the population dynamics of ticks of livestock importance have been determined in Mexico through the seasons of the year and in different states (Estrada-Peña et al., 2006; Alonso-Díaz et al., 2007; González-Cerón et al., 2009; Almazán et al., 2016). For this reason, adequate proposal designs for integrated tick management for all ecological regions of the country are limited.

## Conclusions

EPF have been shown to have good acaricidal effectiveness against ticks of livestock importance and their different biological stages, both in the laboratory and in the field. However, the vast majority of studies have focused on the *R*. *microplus* tick. In accordance with the economic and sanitary importance of other ticks such as *A*. *mixtum* and *R*. *annulatus* in Mexico, it is also recommended to test the efficacy of these fungi against these ticks. Furthermore, the EPF used have been shown to be biologically safe when applied to animals and pastures, including the safety operators. The lack of information on the mechanisms (molecular, genetic, immunological and physiological interactions) involved in the virulence of EPF in ticks was also identified. Most of the information has been obtained on insects, which are taxonomically different from ticks, so these mechanisms may be different. According to the results of the acaricidal efficacy shown by EPF against ticks, they could be considered within an integrated management of these pests. However, it is highly recommended that more studies be carried out on the population dynamics of ticks in the different agroecological regions of the country, more evaluations of tick susceptibility to all available chemical acaricides, and the probable synergy or antagonism of EPF with other alternative control methods, since a paucity of information on these characteristics of ticks and EPF has also been identified.

## Author Contributions

MAA-D: investigation, methodology, writing original draft, supervision, and conceptualization. AF-S: investigation, methodology, writing original draft, supervision, and conceptualization. All authors took part in reviewing and editing of the final manuscript.

## Conflict of Interest

The authors declare that the research was conducted in the absence of any commercial or financial relationships that could be construed as a potential conflict of interest.

## References

[B1] Aboelhadid S. M. Arafa W. M. Mahrous L. N. Fahmy M. M. Kamel A. A. (2018). Molecular detection of *Rhipicephalus* (*Boophilus*) *annulatus* resistance against deltamethrin in middle Egypt. Vet. Parasitol. Reg. Stud. Rep. 13, 198–204. 10.1016/j.vprsr.2018.06.008 31014874

[B2] Adames M. Fernández-Ruvalcaba M. Peña-Chora G. Hernández-Velázquez V. M. (2011). Effects of passages through a suitable host of the fungus, *Metarhizium anisopliae*, on the virulence of acaricide-susceptible and resistant strains of the tick, *Rhipicephalus microplus*. J. Insect. Sci. 11:21. 10.1673/031.011.0121 26983168 PMC4584975

[B3] Aguilar-Domínguez M. Sánchez-Montes S. Esteve-Gassent M. D. Barrientos-Salcedo C. de León A. P. Romero-Salas D. (2019). Genetic structure analysis of *Amblyomma mixtum* populations in Veracruz State, Mexico. Ticks Tick Borne Dis. 10, 86–92. 10.1016/j.ttbdis.2018.09.004 30228080

[B4] Aguilar-Tipacamú G. Nazar P. M. Sesma B. R. Llaven M. Á. O. Martínez C. E. I. Trujillo G. U. B. (2009). Resistencia al amitraz de *Rhipicephalus* (*Boophilus*) *microplus* en unidades de producción del estado de Chiapas. Quehacer Científico en Chiapas 1, 16–22. Availbale online at: https://dgip.unach.mx/images/pdf-REVISTA-QUEHACERCIENTIFICO/QUEHACER-CIENTIFICO-2009-ener-jun/resistencia-al-amitraz_de_rhipicephalus.pdf (accesed November 15, 2020).

[B5] Alcalá-Gómez J. Cruz-Vázquez C. Fernández-Ruvalcaba M. Ángel-Sahagún C. Vitela-Mendoza I. Ramos-Parra M. (2017). Virulence of *Metarhizium anisopliae* and *Beauveria bassiana* isolates and the effects of fungal infection on the reproduction potential of *Rhiphicephalus microplus* engorged females. Biocontrol. Sci. Techn. 27, 931–939. 10.1080/09583157.2017.1366422

[B6] Alegría-López M. A. Rodríguez-Vivas R. I. Torres-Acosta J. F. J. Ojeda-Chi M. M. Rosado-Aguilar J. A. (2015). Use of ivermectin as endoparasiticide in tropical cattle herds generates resistance in gastrointestinal nematodes and the tick *Rhipicephalus microplus* (Acari: Ixodidae). *J. Med. Entomol*. 52, 214–221. 10.1093/jme/tju025 26336306

[B7] Almazán C. Torres-Torres A. Torres-Rodríguez L. Soberanes-Céspedes N. Ortiz-Estrada M. (2016). Aspectos biológicos de *Amblyomma mixtum* (Koch, 1844) en el noreste de México. Quehacer Científico en Chiapas 11, 10–19. Available online at: https://www.dgip.unach.mx/images/pdf-REVISTA-QUEHACERCIENTIFICO/2016-jul-dic/Aspectos_biologicos_de_Amblyomma_mixtum_.pdf (accessed December 1, 2020).

[B8] Alonso-Díaz M. A. Fernández-Salas A. Basurto C. H. (2013b). Manual Técnico: La Garrapata Rhipicephalus (Boophilus) Microplus: su Comportamiento, Control y Resistencia a los Acaricidas en el Trópico Mexicano: 21° D ía del Ganadero. Martínez de la Torre: COFUPRO - FUNPROVER - UNAM. pp. 19–30.

[B9] Alonso-Díaz M. A. Fernández-Salas A. Martínez-Ibáñez F. Osorio-Miranda J. (2013a). *Amblyomma cajennense* (Acari: Ixodidae) tick populations susceptible or resistant to acaricides in the Mexican Tropics. Vet. Parasitol. 197, 326–331. 10.1016/j.vetpar.2013.06.004 23827041

[B10] Alonso-Díaz M. Á. Silva B. J. L. de Magalhães Labarthe A. C. L. Rodríguez-Vivas R. I. (2007). Infestación natural de hembras de *Boophilus microplus* Canestrini, 1887 (Acari: Ixodidae) en dos genotipos de bovinos en el trópico húmedo de Veracruz, México. Vet. Méx. 38, 503–509. Available online at: http://www.redalyc.org/articulo.oa?id=42338410

[B11] Alonso-Díaz M. A. Torres-Acosta J. F. J. Sandoval-Castro C. A. Bruce-Campbell W. (2014). Controlling the introduction and augmentation of parasites in and on domesticated livestock, in Sustainable Food Production Includes Human and Environmental Health, eds W. B. Campbell, and S. López-Ortíz (Netherlands: Springer), 191–228. 10.1007/978-94-007-7454-4_5

[B12] Ángel-Sahagún C. A. Lezama-Gutiérrez R. Molina-Ochoa J. Pescador-Rubio A. Skoda S. R. Cruz-Vázquez C.. (2010). Virulence of Mexican isolates of entomopathogenic fungi (Hypocreales: Clavicipitaceae) upon *Rhipicephalus*= *Boophilus microplus* (Acari: Ixodidae) larvae and the efficacy of conidia formulations to reduce larval tick density under field conditions. Vet. Parasitol. 170, 278–286. 10.1016/j.vetpar.2010.02.037 20359827

[B13] Araújo J. P. Hughes D. P. (2016). Diversity of entomopathogenic fungi: which groups conquered the insect body? Adv. Genet. 94, 1–39. 10.1016/bs.adgen.2016.01.001 27131321

[B14] Araya-Anchetta A. Busch J. D. Scoles G. A. Wagner D. M. (2015). Thirty years of tick population genetics: a comprehensive review. Infect. Genet. Evol. 29, 164–179. 10.1016/j.meegid.2014.11.008 25461844

[B15] Armendáriz-González I. (2003). Informe de un caso de resistencia múltiple a ixodicidas en *Boophilus microplus* Canestrini (Acari: Ixodidae) en Tamaulipas, México. *Vet*. Méx. 34, 397–401. Available online at: http://www.redalyc.org/articulo.oa?id=42334408

[B16] Augustyniuk-Kram A. Kram K. J. (2012). Entomopathogenic fungi as an important natural regulator of insect outbreaks in forests, in Forest ecossistems-more than just trees, ed J. A. Blanco, and L. Yueh-Hsin (Rijeka, Croatia: In Tech Press), 265–294. 10.5772/30596

[B17] Barré N. Uilenberg G. (2010). Spread of parasites transported with their hosts: case study of two species of cattle tick. Rev. sci. tech. Off. int. Epiz. 29, 149–160. 10.20506/rst.29.1.1969 20617654

[B18] Bautista-Gálvez A. B. Segura R. P. Gómez-Vázquez A. (2017). Biological Control of *Rhicephalus* (*Boophilus*) *microplus* with Entomopathogenic Fungi. CIBA Rev. Iberoam. Cienc. Biol. Agropecuarias 6, 33–62. 10.23913/ciba.v6i12.6820359827

[B19] Beys da Silva W. O. Santi L. Corrẽa A. P. F. Silva L. A. D. Bresciani F. R. and Schrank, A. . (2010a). The entomopathogen *Metarhizium anisopliae* can modulate the secretion of lipolytic enzymes in response to different substrates including components of arthropod cuticle. Fungal Biol. 114, 911–916. 10.1016/j.funbio.2010.08.007 21036334

[B20] Beys da Silva W. O. Santi L. Schrank A. Vainstein M. H. (2010b). *Metarhizium anisopliae* lipolytic activity plays a pivotal role in *Rhipicephalus* (*Boophilus*) *microplus* infection. Fungal Biol. 114, 10–15. 10.1016/j.mycres.2009.08.003 20965056

[B21] Beys-da-Silva W. O. Rosa R. L. Berger M. Coutinho-Rodrigues C. J. Vainstein M. H. Schrank A.. (2020). Updating the application of *Metarhizium anisopliae* to control cattle tick *Rhipicephalus microplus* (Acari: Ixodidae). *Exp. Parasitol*. 208:107812. 10.1016/j.exppara.2019.107812 31809704

[B22] Bischoff J. F. Rehner S. A. Humber R. A. (2009). A multilocus phylogeny of the *Metarhizium anisopliae* lineage. Mycologia 101, 512–530. 10.3852/07-202 19623931

[B23] Bram R. A. (1994). Integrated control of ectoparasites. Rev. Off. Int. Epizoot. 13, 1357–1365. 10.20506/rst.13.4.822 7711315

[B24] Brunner-Mendoza C. Reyes-Montes M. D. R. Moonjely S. Bidochka M. J. Toriello C. (2019). A review on the genus *Metarhizium* as an entomopathogenic microbial biocontrol agent with emphasis on its use and utility in Mexico. Biocontrol Sci. Techn. 29, 83–102. 10.1080/09583157.2018.1531111

[B25] Cabrera-Jimenez D. Rodríguez-Vivas R. I. Rosado-Aguilar J. A. (2008). Evaluation of cypermethrin resistance in *Boophilus microplus* strains from cattle farms in the State of Yucatán, Mexico. Téc. Pecu. Méx. 46, 439–447. Available online at: https://cienciaspecuarias.inifap.gob.mx/index.php/Pecuarias/article/view/1793 (accessed November 24, 2020).

[B26] Campos R. A. Arruda W. Boldo J. T. Silva M. V. Barros N. M. Azevedo J. L.. (2005). *Boophilus microplus* infection by *Beauveria amorpha* and *Beauveria bassiana*: SEM analysis and regulation of subtilisin-like proteases and chitinases. Curr. Microbiol. 50, 257–261. 10.1007/s00284-004-4460-y 15886912

[B27] Cárdenas-Canales E. M. Ortega-Santos J. A. Campbell T. A. García-Vázquez Z. Cantú-Covarrubias A. Figueroa-Millán J. V.. (2011). Nilgai antelope in northern Mexico as a possible carrier for cattle fever ticks and *Babesia bovis* and *Babesia bigemina*. J. Wildl. Dis. 47, 777–779. 10.7589/0090-3558-47.3.777 21719852

[B28] Castillo-Gallegos E. de la Mora B. V. Mannetje L. T. Schunemann A. A. (2005). Efecto de introducir *Arachis pintoi* sobre variables del suelo de pasturas de grama nativa del trópico húmedo mexicano. *Téc. Pec*. Méx. 43, 287–295. Available online at: http://www.redalyc.org/articulo.oa?id=61343214

[B29] CFSPH (The Center For Food Security Public Health) (2007). Rhipicephalus (Boophilus) microplus. Iowa State University. P. 3. Available online at: http://www.cfsph.iastate.edu/Factsheets/es/boophilus_microplus-es.pdf (accessed October 24, 2020).

[B30] Chen A. Wang Y. Shao Y. Zhou Q. Chen S. Wu Y.. (2018). Genes involved in *Beauveria bassiana* infection to *Galleria mellonella*. Arch. Microbiol. 200, 541–552. 10.1007/s00203-017-1456-0 29214339

[B31] Cruz-Avalos A. Cruz-Vázquez C. Lezama-Gutiérrez R. Vitela-Mendoza I. Angel-Sahagún C. (2015). Selección de aislados de hongos entomopatógenos para el control de *Rhipicephalus microplus* (Acari: Ixodidae). *Trop. Subtrop. Agroecosyst*. 18, 175–180.

[B32] Cumming G. S. Van Vuuren D. P. (2006). Will climate change affect ectoparasite species ranges? Glob. Ecol. Biogeogr. 15, 486–497. 10.1111/j.1466-822X.2006.00241.x

[B33] De Alva R. (1995). Creating new products for animal health, in Tercer Seminario Internacional de Parasitologia Animal, ed S. Rodríguez-Camarillo, and H. Fragoso-Sánchez (Acapulco), 86–87.

[B34] De Castro J. J. (1997). Sustainable tick and tickborne disease control in livestock improvement in developing countries. Vet. Parasitol. 71, 77–97. 10.1016/S0304-4017(97)00033-2 9261972

[B35] Díaz V. M. Izaguirre F. F. Martínez T. J. J. Aguirre M. J. F. Posada C. S. García C. C. G.. (2014). Efecto de tres cepas de *Metarhizium anisopliae* (Metch) Sor sobre la mortalidad de *Rhipicephalus (Boophilus) microplus* Canestrini en condiciones de laboratorio. Livestock Res. Rural. Dev. 26:2014. Available online at: http://www.lrrd.org/lrrd26/9/diaz26163.html (accessed November 24, 2020).

[B36] Domínguez-García D. D. Agatón F. T. Rosario-Cruz R. (2016). Evaluación económica del control de garrapatas *Rhipicephalus microplus* en México/Economic evaluation of tick (*Rhipicephalus microplus*) control in Mexico. CIBA Rev. Iberoam. Cienc. Biol. Agropecuarias 5, 43–52. 10.23913/ciba.v5i9.49

[B37] Esteve-Gasent M. D. Rodríguez-Vivas R. I. Medina R. F. Ellis D. Schwartz A. Cortés Garcia B.. (2020). Research on integrated management for cattle fever ticks and bovine babesiosis in the United States and Mexico: current status and opportunities for binational coordination. Pathogens 9:871. 10.3390/pathogens9110871 33114005 PMC7690670

[B38] Esteve-Gassent M. D. Pérez de León A. A. Romero-Salas D. Feria-Arroyo T. P. Patino R. Castro-Arellano I.. (2014). Pathogenic landscape of transboundary zoonotic diseases in the Mexico–US border along the Rio Grande. Front. Public. Health. 2:177. 10.3389/fpubh.2014.00177 25453027 PMC4233934

[B39] Estrada-Peña A. (2008). Tick-borne pathogens, transmission rates and climate change. Front. Biosci. 14, 2674–2687. Available online at: https://pdfs.semanticscholar.org/9990/3f0755d290a56049042279ed183025d999a9.pdf (accessed December 4, 2020). 19273227 10.2741/3405

[B40] Estrada-Peña A. García Z. Sánchez H. F. (2006). The distribution and ecological preferences of *Boophilus microplus* (Acari: Ixodidae) in Mexico. Exp. Appl. Acarol. 38, 307–316. 10.1007/s10493-006-7251-2 16612672

[B41] Estrada-Peña A. Guglielmone A. A. Mangold A. J. (2004). The distribution and ecological 'preferences' of the tick *Amblyomma cajennense* (Acari: Ixodidae), an ectoparasite of humans and other mammals in the Americas. Ann. Trop. Med. Parasitol. 98, 283–292. 10.1179/000349804225003316 15119974

[B42] Estrada-Peña A. Venzal J. M. (2006). High-resolution predictive mapping for *Boophilus annulatus* and *B. microplus* (Acari: Ixodidae) in Mexico and Southern Texas. Vet. Parasitol. 14, 350–358. 10.1016/j.vetpar.2006.07.003 16956729

[B43] Falvey J. L. (2015). Food security: the contribution of livestock. Chiang Mai Univ. J. Nat. Sci. 14, 103–117. 10.12982/CMUJNS.2015.0074

[B44] Fang W. Leng B. Xiao Y. Jin K. Ma J. Fan Y.. (2005). Cloning of *Beauveria bassiana* chitinase gene *Bbchit1* and its application to improve fungal strain virulence. Appl. Environ. Microbiol. 71, 363–370. 10.1128/AEM.71.1.363-370.2005 15640210 PMC544255

[B45] Fernandes É. K. Bittencourt V. R. Roberts D. W. (2012). Perspectives on the potential of entomopathogenic fungi in biological control of ticks. Exp. Parasitol. 130, 300–305. 10.1016/j.exppara.2011.11.004 22143088

[B46] Fernandes É. K. K. Bittencourt V. R. E. P. (2008). Entomopathogenic fungi against South American tick species, in Diseases of Mites and Ticks, eds J. Bruin, and L. P. S. van der Geest (Dordrecht: Springer), 71–93. 10.1007/978-1-4020-9695-2_8 18563593

[B47] Fernández-Ruvalcaba M. Zhioua E. García-Vázquez Z. (2005). Infectividad de *Metarhizium anisopliae* en contra de cepas de garrapata *Boophilus microplus* sensible y resistente a los organofosforados. Téc. Pecu. Méx. 43, 433–440. Available online at: http://www.redalyc.org/articulo.oa?id=61343313

[B48] Fernández-Salas A. Alonso-Díaz M. A. Alonso-Morales R. A. (2019). Effect of entomopathogenic native fungi from paddock soils against *Rhipicephalus microplus* larvae with different toxicological behaviors to acaricides. Exp. Parasitol. 204:107729. 10.1016/j.exppara.2019.107729 31348914

[B49] Fernández-Salas A. Alonso-Díaz M. A. Alonso-Morales R. A. Lezama-Gutiérrez R. Rodríguez-Rodríguez J. C. Cervantes-Chávez J. A. (2017). Acaricidal activity of *Metarhizium anisopliae* isolated from paddocks in the Mexican tropics against two populations of the cattle tick *Rhipicephalus microplus*. Med. Vet. Entomol. 31, 36–43. 10.1111/mve.12203 27759176

[B50] Fernández-Salas A. Alonso-Díaz M. Á. Morales R. A. A. Lezama-Gutiérrez R. Cervantes-Chávez J. A. (2018). Phylogenetic relationships and acaricidal effects of *Beauveria bassiana* obtained from cattle farm soils against *Rhipicephalus microplus*. J. Parasitol. 104, 275–282. 10.1645/17-162 29457960

[B51] Fernandez-Salas A. Alonso-Morales R. A. Alonso-Díaz M. Á. (2020). Distribution of entomopathogenic fungi in soils of cattle farms and factors associated with their presence in the Mexican tropics. *Trop. Subtrop. Agroecosyst*. 23, 1–12. Available online at: http://www.revista.ccba.uady.mx/urn:ISSN:1870-0462-tsaes.v23i3.3293

[B52] Fernández-Salas A. Rodríguez-Vivas R. I. Alonso-Díaz M. Á. (2012a). Resistance of *Rhipicephalus microplus* to amitraz and cypermethrin in tropical cattle farms in Veracruz, Mexico. J. Parasitol. 98, 1010–1014. 10.1645/GE-3074.1 22524292

[B53] Fernández-Salas A. Rodríguez-Vivas R. I. Alonso-Díaz M. A. (2012c). First report of a *Rhipicephalus microplus* tick population multi-resistant to acaricides and ivermectin in the Mexican tropics. Vet. Parasitol. 183, 338–342. 10.1016/j.vetpar.2011.07.028 21824728

[B54] Fernández-Salas A. Rodríguez-Vivas R. I. Alonso-Díaz M. A. Basurto-Camberos H. (2012b). Ivermectin resistance status and factors associated in *Rhipicephalus microplus* (Acari: Ixodidae) populations from Veracruz, Mexico. Vet. Parasitol. 190, 210–215. 10.1016/j.vetpar.2012.06.003 22785128

[B55] Freimoser F. M. Hu G. St Leger R. J. (2005). Variation in gene expression patterns as the insect pathogen *Metarhizium anisopliae* adapts to different host cuticles or nutrient deprivation *in vitro*. Microbiology 151, 361–371. 10.1099/mic.0.27560-0 15699187

[B56] Gao B. J. Mou Y. N. Tong S. M. Ying S. H. Feng M. G. (2020). Subtilisin-like Pr1 proteases marking the evolution of pathogenicity in a wide-spectrum insect-pathogenic fungus. Virulence 11, 365–380. 10.1080/21505594.2020.1749487 32253991 PMC7199741

[B57] Giles J. R. Peterson A. T. Busch J. D. Olafson P. U. Scoles G. A. Davey R. B.. (2014). Invasive potential of cattle fever ticks in the southern United States. Parasites Vectors 7:189. 10.1186/1756-3305-7-189 24742062 PMC4021724

[B58] Goettel M. S. Johnson D. L. (1992). Environmental Impact and Safety of Fungal Biocontrol Agents. Available online at: https://agris.fao.org/agris-search/search.do?recordID=GB9124449 (accessed January 7, 2021).

[B59] Golo P. S. Angelo I. C. Camargo M. G. Perinotto W. M. S. Bittencourt V. R. E. P. (2011). Effects of destruxin A on *Rhipicephalus (Boophilus) microplus* ticks (Acari: Ixodidae). *Rev. Bras. Parasitol. Vet*. 20, 338–341. 10.1590/S1984-29612011000400015 22166391

[B60] Golo P. S. Santos H. A. Perinotto W. M. Quinelato S. Angelo I. C. Camargo M. G.. (2015). The influence of conidial Pr1 protease on pathogenicity potential of *Metarhizium anisopliae* senso latu to ticks. Parasitol. Res. 114, 2309–2315. 10.1007/s00436-015-4426-y 25786608

[B61] González-Cerón F. Becerril-Pérez C. M. Torres-Hernández G. Díaz-Rivera P. Santellano-Estrada E. Rosendo-Ponce A. (2009). Infestación natural por *Amblyomma cajennense* y *Boophilus microplus* en bovinos criollo lechero tropical durante la época de lluvias. Agrociencia 43, 577–584. Available online at: http://www.scielo.org.mx/scielo.php?script=sci_arttext&pid=S1405-31952009000600003 (accessed April 17, 2021).

[B62] González-Padilla E. Lassala A. Pedernera M. Gutierrez C. G. (2019). Cow-calf management practices in Mexico: farm organization and infrastructure. Vet. Méx. 6, 1–17. 10.22201/fmvz.24486760e.2019.3.677

[B63] Grisi L. Leite R. C. Martins J. R. D. S. Barros A. T. M. D. Andreotti R. Cançado P. H. D.. (2014). Reassessment of the potential economic impact of cattle parasites in Brazil. Rev. Bras. Parasitol. Vet. 23, 150–156. 10.1590/S1984-29612014042 25054492

[B64] Higa L. D. O. S. Piña F. T. B. da Silva Rodrigues V. Garcia M. V. Salas D. R. Miller R. J.. (2020). Evidence of acaricide resistance in different life stages of *Amblyomma mixtum* and *Rhipicephalus microplus* (Acari: Ixodidae) collected from the same farm in the state of Veracruz, Mexico. Prev. Vet. Med. 174:104837. 10.1016/j.prevetmed.2019.104837 31756672

[B65] Humber R. A. (2008). Evolution of entomopathogenicity in fungi. J. Invert. Pathol. 98, 262–266. 10.1016/j.jip.2008.02.017 18423482

[B66] Jaber L. R. Ownley B. H. (2018). Can we use entomopathogenic fungi as endophytes for dual biological control of insect pests and plant pathogens? Biol. Control. 116, 36–45. 10.1016/j.biocontrol.2017.01.018

[B67] Jiménez-Ruíz M. (2015). Efecto acaricida in vitro de hongos entomopatógenos nativos de Veracruz, México, contra larvas de garrapata Amblyomma cajennense (Acari: Ixodidae) (Dissertation/Bachelor's thesis). Oaxaca: Universidad Autónoma Benito Juarez de Oaxaca.

[B68] Kirkland B. H. Cho E. M. Keyhani O. N. (2004). Differential susceptibility of *Amblyomma maculatum* and *Amblyomma americanum* (Acari: Ixodidea) to the entomopathogenic fungi *Beauveria bassiana* and *Metarhizium anisopliae*. Biol. Control. 31, 414–421. 10.1016/j.biocontrol.2004.07.007

[B69] Kiss T. Cadar D. Spînu M. (2012). Tick prevention at a crossroad: new and renewed solutions. Vet. Parasitol. 187, 357–366. 10.1016/j.vetpar.2012.02.010 22424918

[B70] Klafke G. M. Moreno H. C. Tidwell J. P. Miller R. J. Thomas D. B. Feria-Arroyo T. P.. (2020). Partial characterization of the voltage-gated sodium channel gene and molecular detection of permethrin resistance in *Rhipicephalus annulatus* (Say, 1821). *Ticks Tick Borne Dis*. 11:101368. 10.1016/j.ttbdis.2019.101368 31917128

[B71] Kutz S. Jenkins E. Veitch A. Ducrocq J. Polley L. Elkin B. (2009). The Arctic as a model for anticipating, preventing, and mitigating climate change impacts on host–parasite interactions. Vet. Parasitol. 163, 217–228. 10.1016/j.vetpar.2009.06.008 19560274

[B72] Li A. Y. Davey R. B. Miller R. J. George J. E. (2003). Resistance to coumaphos and diazinon in *Boophilus microplus* (Acari: Ixodidae) and evidence for the involvement of an oxidative detoxification mechanism. J. Med. Entomol. 40, 482–490. 10.1603/0022-2585-40.4.482 14680115

[B73] Li A. Y. Davey R. B. Miller R. J. George J. E. (2004). Detection and characterization of amitraz resistance in the southern cattle tick, *Boophilus microplus* (Acari: Ixodidae). *J. Med. Entomol*. 41, 193–200. 10.1603/0022-2585-41.2.193 15061278

[B74] Lohmeyer K. H. Pound J. M. May M. A. Kammlah D. M. Davey R. B. (2011). Distribution of *Rhipicephalus (Boophilus) microplus* and *Rhipicephalus (Boophilus) annulatus* (Acari: Ixodidae) infestations detected in the United States along the Texas/Mexico border. J. Med. Entomol. 48, 770–774. 10.1603/ME10209 21845935

[B75] Maina U. M. Galadima I. B. Gambo F. M. Zakaria D. (2018). A review on the use of entomopathogenic fungi in the management of insect pests of field crops. J. Entomol. Zool. Stud. 6, 27–32. Available online at: https://www.entomoljournal.com/archives/2018/vol6issue1/PartA/5-5-367-216.pdf

[B76] Mantzoukas S. Eliopoulos P. A. (2020). Endophytic entomopathogenic fungi: a valuable biological control tool against plant pests. Appl. Sci. 10:360. 10.3390/app10010360

[B77] Martínez I. F. De Labra V. G. Osorio M. J. (2019). Toxicological response of different genera and species of ixodide ticks collected in Mexico, in Memorias del XI Congreso Nacional de Parasitología Veterinaria (Monterrey), 139–145.

[B78] Merino O. De la Cruz N. I. Martinez J. de León A. P. Romero-Salas D. Esteve-Gassent M. D.. (2020). Molecular detection of Rickettsia species in ticks collected in the Mexico-USA transboundary region. Exp. Appl. Acarol. 80, 559–567. 10.1007/s10493-020-00483-5 32249393

[B79] Miller R. J. Almazán C. Ortíz-Estrada M. Davey R. B. George J. E. De León A. P. (2013). First report of fipronil resistance in *Rhipicephalus* (*Boophilus*) *microplus* of Mexico. Vet. Parasitol. 191, 97–101. 10.1016/j.vetpar.2012.08.011 23026557

[B80] Miller R. J. Li A. Y. Tijerina M. Davey R. B. George J. E. (2008). Differential response to diazinon and coumaphos in a strain of *Boophilus microplus* (Acari: Ixodidae) collected in Mexico. J. Med. Entomol. 45, 905–911. 10.1603/0022-2585(2008)45[905:DRTDAC]2.0.CO;2 18826034

[B81] Miller R. J. Rentaria J. A. E. Martinez H. Q. George J. E. (2007). Characterization of permethrin-resistant *Boophilus microplus* (Acari: Ixodidae) collected from the state of Coahuila, Mexico. J. Med. Entomol. 44, 895–897. 10.1603/0022-2585(2007)44[895:COPBMA]2.0.CO.CO;2 17915523

[B82] Montero E. González L. M. Chaparro A. Benzal J. Bertellotti M. Masero J. A.. (2016). First record of *Babesia* sp. in Antarctic penguins. Ticks. Tick. Borne. Dis. 7, 498–501. 10.1016/j.ttbdis.2016.02.006 26874670

[B83] Mora M. A. E. Castilho A. M. C. Fraga M. E. (2017). Classification and infection mechanism of entomopathogenic fungi. Arq. Inst. Biol. 84:e0552015. 10.1590/1808-1657000552015

[B84] Morais-Urano R. P. Chagas A. C. Berlinck R. G. (2012). Acaricidal action of destruxins produced by a marine-derived Beauveria felina on the bovine tick Rhipicephalus (Boophilus) microplus. *Exp*. Parasitol. 132, 362–366. 10.1016/j.exppara.2012.08.011 22955115

[B85] Morillón D. Silva Casarín R. Valdés H. P. (2018). Atlas del Impacto del Océano en el Clima en México. Campeche: Cemie-Océano, Universidad Autónoma de Campeche. p. 128.

[B86] NASA-GCC (2019). Overview: Weather, Global Warming and Climate Change. Global Warming vs Climate Change. Resource 32. Available online at: https://climate.nasa.gov/resources/global-warming-vs-climate-change/ (accessed December 14, 2020).

[B87] Needham G. R. Teel P. D. (1991). Off-host physiological ecology of ixodid ticks. Ann. Rev. Entomol. 36, 659–681. 10.1146/annurev.en.36.010191.003303 2006871

[B88] Ojeda-Chi M. M. Rodríguez-Vivas R. I. Galindo-Velasco E. Lezama-Gutiérrez R. Cruz-Vázquez C. (2011). Control de *Rhipicephalus microplus* (Acari: Ixodidae) mediante el uso del hongo entomopatógeno *Metarhizium anisopliae* (Hypocreales: Clavicipitaceae): Revisión. Rev. Mex. Cienc. Pec. 2, 177–192. Available online at: http://www.scielo.org.mx/scielo.php?script=sci_arttext&pid=S2007-11242011000200005

[B89] Ojeda-Chi M. M. Rodriguez-Vivas R. I. Galindo-Velasco E. Lezama-Gutiérrrez R. (2010). Laboratory and field evaluation of *Metarhizium anisopliae* (Deuteromycotina: Hyphomycetes) for the control of *Rhipicephalus microplus* (Acari: Ixodidae) in the Mexican tropics. Vet. Parasitol. 170, 348–354. 10.1016/j.vetpar.2010.02.022 20299149

[B90] Omkar K. K. (2016). Ecofriendly Pest Management for Food Security. Cambridge, MA: Elsevier.

[B91] Ortiz E. M. Santamaría V. M. Ortiz N. A. Soberanes C. N. Osorio M. J. Franco B. R.. (1995). Caracterización de la resistencia de *Boophilus microplus* a ixodicidas en México, in Memorias de IV Seminario Internacional de Parasitologí a Animal, Resistencia y Control en Garrapatas y Moscas de Importancia Veterinaria (Acapulco), 58–66

[B92] Ortiz-Urquiza A. Keyhani N. O. (2013). Action on the surface: Entomopathogenic fungi versus the insect cuticle. Insects 4, 357–374. 10.3390/insects4030357 26462424 PMC4553469

[B93] Pérez de León A. A. Mitchell R. D. Watson D. W. (2020). Ectoparasites of cattle. Vet. Clin. North Am. Food Anim. Pract. 36, 173–185. 10.1016/j.cvfa.2019.12.004 32029183

[B94] Pérez de León A. A. Teel P. D. Auclair A. N. Messenger M. T. Guerrero F. D. Schuster G.. (2012). Integrated strategy for sustainable cattle fever tick eradication in USA is required to mitigate the impact of global change. Front. Physiol. 3:195. 10.3389/fphys.2012.00195 22712018 PMC3374960

[B95] Perez-Cogollo L. C. Rodriguez-Vivas R. I. Ramirez-Cruz G. T. Miller R. J. (2010a). First report of the cattle tick *Rhipicephalus microplus* resistant to ivermectin in Mexico. Vet. Parasitol. 168, 165–169. 10.1016/j.vetpar.2009.10.021 19951828

[B96] Perez-Cogollo L. C. Rodriguez-Vivas R. I. Ramirez-Cruz G. T. Rosado-Aguilar J. A. (2010b). Survey of *Rhipicephalus microplus* resistance to ivermectin at cattle farms with history of macrocyclic lactones use in Yucatan, Mexico. Vet. Parasitol. 172, 109–113. 10.1016/j.vetpar.2010.04.030 20570047

[B97] Perinotto W. M. S. Angelo I. C. Golo P. S. Quinelato S. Camargo M. G. Sá F. A.. (2012). Susceptibility of different populations of ticks to entomopathogenic fungi. Exp. Parasitol. 130, 257–260. 10.1016/j.exppara.2011.12.003 22212684

[B98] Polar P. Kairo M. T. Peterkin D. Moore D. Pegram R. John S. A. (2005). Assessment of fungal isolates for development of a myco-acaricide for cattle tick control. Vector Borne Zoonotic Dis. 5, 276–284. 10.1089/vbz.2005.5.276 16187897

[B99] Polley L. Thompson A. (2009). Parasite zoonoses and climate change: molecular tools for tracking shifting boundaries. Trends Parasitol. 25, 285–291. 10.1016/j.pt.2009.03.007 19428303

[B100] Porfirio N. I. Schwentesius R. R. (2016). Control Biológico de Garrapata (*Boophilus microplus*), *con Microorganismos*. Available online at: https://www.researchgate.net/profile/Rita_Rindermann/publication/289768055_Control_biologico_de_Garrapata_con_Microorganismos/links/569282aa08aed0aed8167e2b.pdf (accessed April 17, 2021).

[B101] Pound J. M. George J. E. Kammlah D. M. Lohmeyer K. H. Davey R. B. (2010). Evidence for role of white-tailed deer (Artiodactyla: Cervidae) in epizootiology of cattle ticks and southern cattle ticks (Acari: Ixodidae) in reinfestations along the Texas/Mexico border in south Texas: a review and update. J. Econ. Entomol. 103, 211–218. 10.1603/EC09359 20429430

[B102] Prieto G. (2005). El Clima de México a Través de los Mapas. Geografía infinita. Available online at: https://www.geografiainfinita.com/2015/07/el-clima-de-mexico-a-traves-de-los-mapas/ (accessed March 3, 2021).

[B103] Racelis A. E. Davey R. B. Goolsby J. A. De León A. P. Varner K. Duhaime R. (2012). Facilitative ecological interactions between invasive species: arundo donax stands as favorable habitat for cattle ticks (Acari: Ixodidae) along the US–Mexico border. J. Med. Entomol. 49, 410–417. 10.1603/ME11104 22493861

[B104] Rajula J. Rahman A. Krutmuang P. (2020). Entomopathogenic fungi in Southeast Asia and Africa and their possible adoption in biological control. Biol. Control. 151:104399. 10.1016/j.biocontrol.2020.104399

[B105] Rehner S. A. Minnis A. M. Sung G. H. Luangsa-ard J. J. Devotto L. Humber R. A. (2011). Phylogeny and systematics of the anamorphic, entomopathogenic genus *Beauveria*. Mycol. 103, 1055–1073. 10.3852/10-302 21482632

[B106] Rivera-Cervantes M. Vargas-Sandoval M. Ramos-Lima M. de Jesús J. Ayala-Ortega M. Lara-Chávez B. N.. (2017). Acción de *Metarhizuim anisopliae* (Metschnikoff) en *Rhipicephalus* (*B*.) *microplus* (Canestrini) (Acari: Ixodidae) sobre ganado bovino en Coalcomán, Michoacán. Entomol. Mexicana 4, 213–219. Available online at: http://entomologia.socmexent.org/revista/2017/CB/EM2792017_213-219.pdf (accessed January 2, 2021).

[B107] Rodríguez-Alcocer U. J. Rodríguez-Vivas R. I. Ojeda-Chi M. M. Galindo-Velasco E. Lezama-Gutiérrez R. (2014). Eficacia de la mezcla de dos cepas de *Metarhizium anisopliae* (Deuteromycotina: Hyphomycetes) para el control de *Rhipicephalus microplus* en infestaciones naturales en bovinos. *Trop. Subtrop*. Agroecosyst. 17, 223–229. Available online at: http://www.redalyc.org/articulo.oa?id=93931761008

[B108] Rodriguez-Vivas R. I. Alonso-Díaz M. A. Rodríguez-Arevalo F. Fragoso-Sanchez H. Santamaria V. M. Rosario-Cruz R. (2006a). Prevalence and potential risk factors for organophosphate and pyrethroid resistance in *Boophilus microplus* ticks on cattle ranches from the state of Yucatan, Mexico. Vet. Parasitol. 136, 335–342. 10.1016/j.vetpar.2005.05.069 16413971

[B109] Rodríguez-Vivas R. I. Grisi L. de León A. A. P. Villela H. S. de Jesús Torres-Acosta J. F. Sánchez H. F.. (2017). Potential economic impact assessment for cattle parasites in Mexico. Review. Rev. Mex. Cienc. Pecuarias. 8, 61–74. 10.22319/rmcp.v8i1.4305

[B110] Rodriguez-Vivas R. I. Hodgkinson J. E. Rosado-Aguilar J. A. Villegas-Perez S. L. Trees A. J. (2012). The prevalence of pyrethroid resistance phenotype and genotype in *Rhipicephalus* (*Boophilus*) *microplus* in Yucatan, Mexico. Vet. Parasitol. 184,221–229. 10.1016/j.vetpar.2011.09.017 21978740

[B111] Rodriguez-Vivas R. I. Jonsson N. N. Bhushan C. (2018). Strategies for the control of *Rhipicephalus microplus* ticks in a world of conventional acaricide and macrocyclic lactone resistance. Parasitol. Res. 117, 3–29. 10.1007/s00436-017-5677-6 29152691 PMC5748392

[B112] Rodríguez-Vivas R. I. Li A. Y. Ojeda-Chi M. M. Trinidad-Martinez I. Rosado-Aguilar J. A. Miller R. J.. (2013b). *In vitro* and *in vivo* evaluation of cypermethrin, amitraz, and piperonyl butoxide mixtures for the control of resistant *Rhipicephalus* (*Boophilus*) *microplus* (Acari: Ixodidae) in the Mexican tropics. Vet. Parasitol. 197, 288–296. 10.1016/j.vetpar.2013.07.018 23948559

[B113] Rodríguez-Vivas R. I. Ojeda-Chi M. M. Rosado-Aguilar J. A. Trinidad-Martínez I. C. Torres-Acosta J. F. J. Ticante-Perez V.. (2013a). Red deer (*Cervus elaphus*) as a host for the cattle tick *Rhipicephalus microplus* (Acari: Ixodidae) in Yucatan, Mexico. Exp. Appl. Acarol. 60, 543–552. 10.1007/s10493-013-9672-z 23423423

[B114] Rodríguez-Vivas R. I. Pérez-Cogollo L. C. Rosado-Aguilar J. A. Ojeda-Chi M. M. Trinidad-Martinez I. Miller R. J.. (2014a). Rhipicephalus (Boophilus) microplus resistant to acaricides and ivermectin in cattle farms of Mexico. Braz. J. Vet. Parasitol. 23, 113–122. 10.1590/S1984-29612014044 25054487

[B115] Rodríguez-Vivas R. I. Quiñones A. F. Fragoso-Sánchez H. (2005). epidemiolgía y control de la garrapata *Boophilus* en México, in Enfermedades de Importancia Económica en Producción Animal, ed R. I. Rodríguez-Vivas (México, DF: McGraw-Hill-UADY), 571–592.

[B116] Rodriguez-Vivas R. I. Rivas A. L. Chowell G. Fragoso S. H. Rosario C. R. Garcia Z.. (2007). Spatial distribution of acaricide profiles (*Boophilus microplus* strains susceptible or resistant to acaricides) in southeastern Mexico. Vet. Parasitol. 146, 158–169. 10.1016/j.vetpar.2007.01.016 17349747

[B117] Rodriguez-Vivas R. I. Rodriguez-Arevalo F. Alonso-Díaz M. A. Fragoso-Sanchez H. Santamaria V. M. Rosario-Cruz R. (2006b). Prevalence and potential risk factors for amitraz resistance in *Boophilus microplus* ticks in cattle farms in the State of Yucatan, Mexico. Prev. Vet. Med. 75, 280–286. 10.1016/j.prevetmed.2006.04.001 16730819

[B118] Rodríguez-Vivas R. I. Rosado-Aguilar J. A. Ojeda-Chi M. M. Pérez-Cogollo L. C. Trinidad-Martínez I. Bolio-González M. E. (2014b). Control integrado de garrapatas en la ganadería bovina. Ecosistemas Recur. Agropecuarios 1, 295–308.

[B119] Romero-Pérez J. B. (2020). Aislamiento e identificación de hongos entomopatógenos y su evaluación garrapaticida in vitro mediante manipulación de su desarrollo en diferentes sustratos (Dissertation/Bachelor's thesis). Xicotepetl: Universidad de Xicotepetl.

[B120] Romo-Martínez A. Fernández-Ruvalcaba M. Hernández-Velázquez V. M. Peña-Chora G. Lina-García L. P. Osorio-Miranda J. (2013). Evaluation of natural origin products for the control of *Rhipicephalus* (*Boophilus*) *microplus* (Acari: Ixodidae) on cattle artificially infested. *Basic. Res. J. Agric. Sci*. Rev. 2, 64–79. Available online at: https://www.researchgate.net/publication/280309624_Evaluation_of_natural_origin_products_for_the_control_of_Rhipicephalus_Boophilus_microplus_Acari_Ixodidae_on_cattle_artificially_infested

[B121] Rosado-Aguilar J. A. Rodriguez-Vivas R. I. Garcia-Vazquez Z. Fragoso-Sanchez H. Ortiz-Najera A. Rosario-Cruz R. (2008). Development of amitraz resistance in field populations of *Boophilus microplus* (Acari: Ixodidae) undergoing typical amitraz exposure in the Mexican tropics. Vet. Parasitol. 152:349–353. 10.1016/j.vetpar.2007.12.026 18242859

[B122] Rosario-Cruz R. Guerrero F. D. Miller R. J. Rodriguez-Vivas R. I. Tijerina M. Dominguez-Garcia D. I.. (2009). Molecular survey of pyrethroid resistance mechanisms in Mexican field populations of *Rhipicephalus (Boophilus) microplus*. Parasitol. Res. 105, 1145–1153. 10.1007/s00436-009-1539-1 19565267 PMC2729983

[B123] Samish M. Ginsberg H. Glazer I. (2004). Biological control of ticks. Parasitology 129:S389. 10.1017/S0031182004005219 15938520

[B124] Sánchez-Montes D. Ríos-Muñoz C. Espinoza-Martínez D. Guzmán-Cornejo C. Berzunza-Cruz M. Becker I. (2016). First Report of *Candidatus Rickettsia amblyommii* in West Coast of México. Ticks Tick. Borne. Dis. 7, 1139–1145. 10.1016/j.ttbdis.2016.08.007 27616774

[B125] Schrank A. Vainstein M. H. (2010). *Metarhizium anisopliae* enzymes and toxins. Toxicon 56, 1267–1274. 10.1016/j.toxicon.2010.03.008 20298710

[B126] SEMARNAT (Secretaría de Medio Ambiente y Recursos Naturales). (2003). Atlas Digital Geográfico. Climas de México. Available online at: http://gisviewer.semarnat.gob.mx/aplicaciones/Atlas2015/atm_climas.html (accessed March 10, 2021).

[B127] Semenova T. A. Dunaevsky Y. E. Beljakova G. A. Belozersky M. A. (2020). Extracellular peptidases of insect-associated fungi and their possible use in biological control programs and as pathogenicity markers. Fungal Biol. 124, 65–72. 10.1016/j.funbio.2019.11.005 31892378

[B128] SENASICA (2013). Distribución y Diversidad de la Garrapata Boophilus spp. SAGARPA-Boletí*n Técnico*. Available online at: https://www.aphis.usda.gov/import_export/downloads/presentations/Boophilus-tick.pdf (accessed January, 5, 2021).

[B129] SENASICA-SAGARPA (2015). Distribución y Diversidad de la Garrapatas Boophilus spp. Available online at: https://www.aphis.usda.gov/import_export/downloads/presentations/Boophilus-tick.pdf (accessed March 1, 2021).

[B130] SIAP (Servicio de Información Agroalimentaria y Pesquera) (2018). La ganaderí*a: S*í*mbolo de Fortaleza del Campo Mexicano*. Día Nacional de la Ganadería. Gobierno de México. Available online at: https://www.gob.mx/siap/articulos/la-ganaderia-simbolo-de-fortaleza-del-campo-mexicano (accessed December 4, 2020).

[B131] SIAP (Servicio de Información Agroalimentaria y Pesquera) (2020). La Ganaderí*a: Sustento de la Econom*í*a y Vida del Pa*í*s*. Día de la ganadería en México. SADER-CDMX. Available online at: https://www.gob.mx/agricultura/cdmx/articulos/dia-de-la-ganaderia-en-mexico?idiom=es (accessed December 11, 2020).

[B132] Skinner M. Parker B. L. Kim J. S. (2014). Role of entomopathogenic fungi in integrated pest management. J. Integ. Pest. Manag. 10, 169–191. 10.1016/B978-0-12-398529-3.00011-7

[B133] Snelson J. T. (1975). Animal ectoparasites and disease vector causing major reduction in world food supplies. FAO Plant Prot. Bull. 13, 103–114.

[B134] Soberanes N. C. Vargas M. S. Sánchez H. F. Vázquez Z. G. (2002). Primer caso de resistencia al amitraz en la garrapata del ganado *Boophilus microplus* en México. Téc. Pec. Méx. 40, 81–92. Available online at: http://www.redalyc.org/articulo.oa?id=61340106

[B135] Sousa L. A. D. Pires Júnior H. B. Soares S. F. Ferri P. H. Ribas P. Lima E. M.. (2011). Potential synergistic effect of *Melia azedarach* fruit extract and *Beauveria bassiana* in the control of *Rhipicephalus* (*Boophilus*) *microplus* (Acari: Ixodidae) in cattle infestations. Vet. Parasitol. 175, 320–324. 10.1016/j.vetpar.2010.10.012 21055878

[B136] Srinivasan R. Sevgan S. Ekesi S. Tamó M. (2019). Biopesticide based sustainable pest management for safer production of vegetable legumes and brassicas in Asia and Africa. Pest. Manag. Sci. 75, 2446–2454. 10.1002/ps.5480 31074055

[B137] St. Leger R. J. Staples R. C. Roberts D. W. (1992). Cloning and regulatory analysis of starvation-stress gene, ssgA, encoding a hydrophobin-like protein from the entomopathogenic fungus, *Metarhizium anisopliae*. Gene 120, 119–124. 10.1016/0378-1119(92)90019-L 1398117

[B138] Valero-Jiménez C. A. Wiegers H. Zwaan B. J. Koenraadt C. J. van Kan J. A. (2016). Genes involved in virulence of the entomopathogenic fungus *Beauveria bassiana*. J. Invertebr. Pathol. 133, 41–49. 10.1016/j.jip.2015.11.011 26628209

[B139] Wang C. St Leger R. J. (2007). The MAD1 adhesin of *Metarhizium anisopliae* links adhesion with blastospore production and virulence to insects, and the MAD2 adhesin enables attachment to plants. Eukaryotic Cell. 6, 808–816. 10.1128/EC.00409-06 17337634 PMC1899246

[B140] Webb S. L. Demarais S. Zaiglin R. E. Pollock M. T. Whittaker D. G. (2010). Size and fidelity of home ranges of male white-tailed deer (Odocoileus virginianus) in southern Texas. Southw. Naturalist. 55, 269–273. 10.1894/TAL-10.1

[B141] Webster A. Pradel E. Souza U. A. Martins J. R. Reck J. Schrank A.. (2017). Does the effect of a *Metarhizium anisopliae* isolate on *Rhipicephalus microplus* depend on the tick population evaluated? Ticks. Tick. Borne. Dis. 8, 270–274. 10.1016/j.ttbdis.2016.11.012 27908773

[B142] Webster A. Reck J. Santi L. Souza U.A. Dall'Agnol B. Klafke G.M.. (2105). Integrated control of an acaricide-resistant strain of the cattle tick *Rhipicephalus microplus* by applying *Metarhizium anisopliae* associated with cypermethrin and chlorpyriphos under field conditions. Vet. Parasitol. 207, 302–308. 10.1016/j.vetpar.2014.11.021 25577676

[B143] Wong H. J. Mohamad-Fauzi N. Rizman-Idid M. Convey P. Alias S. A. (2019). Protective mechanisms and responses of micro-fungi towards ultraviolet- induced cellular damage. Polar Sci. 20, 19–34. 10.1016/j.polar.2018.10.001

[B144] Ziapour S. P. Kheiri S. Fazeli-Dinan M. Sahraei-Rostami F. Mohammadpour R. A. Aarabi M.. (2017). Pyrethroid resistance in Iranian field populations of *Rhipicephalus* (*Boophilus*) *annulatus*. Pestic. Biochem. Physiol. 136, 70–79. 10.1016/j.pestbp.2016.08.001 28187834

[B145] Zimmermann G. (2007). Review on safety of the entomopathogenic fungi *Beauveria bassiana* and *Beauveria brongniartii*. Biocontrol. Sci. Tech. 17, 553–596. 10.1080/09583150701309006

